# Screening Pyridine Derivatives against Human Hydrogen Sulfide-synthesizing Enzymes by Orthogonal Methods

**DOI:** 10.1038/s41598-018-36994-w

**Published:** 2019-01-24

**Authors:** Karim Zuhra, Pedro M. F. Sousa, Giulia Paulini, Ana Rita Lemos, Zenta Kalme, Imants Bisenieks, Egils Bisenieks, Brigita Vigante, Gunars Duburs, Tiago M. Bandeiras, Luciano Saso, Alessandro Giuffrè, João B. Vicente

**Affiliations:** 1 Instituto de Tecnologia Química e Biológica António Xavier, Universidade Nova de Lisboa, Oeiras, Portugal; 2 CNR Institute of Molecular Biology and Pathology, Rome, Italy; 3 Department of Biochemical Sciences, Sapienza University of Rome, Rome, Italy; 4 Instituto de Biologia Experimental e Tecnológica, Oeiras, Portugal; 5 Latvian Institute of Organic Synthesis, Riga, Latvia; 6 Department of Physiology and Pharmacology “Vittorio Erspamer”, Sapienza University of Rome, Rome, Italy

## Abstract

Biosynthesis of hydrogen sulfide (H_2_S), a key signalling molecule in human (patho)physiology, is mostly accomplished by the human enzymes cystathionine β-synthase (CBS), cystathionine γ-lyase (CSE) and 3-mercaptopyruvate sulfurtransferase (MST). Several lines of evidence have shown a close correlation between increased H_2_S production and human diseases, such as several cancer types and amyotrophic lateral sclerosis. Identifying compounds selectively and potently inhibiting the human H_2_S-synthesizing enzymes may therefore prove beneficial for pharmacological applications. Here, the human enzymes CBS, CSE and MST were expressed and purified from *Escherichia coli*, and thirty-one pyridine derivatives were synthesized and screened for their ability to bind and inhibit these enzymes. Using differential scanning fluorimetry (DSF), surface plasmon resonance (SPR), circular dichroism spectropolarimetry (CD), and activity assays based on fluorimetric and colorimetric H_2_S detection, two compounds (C30 and C31) sharing structural similarities were found to weakly inhibit both CBS and CSE: 1 mM C30 inhibited these enzymes by approx. 50% and 40%, respectively, while 0.5 mM C31 accounted for CBS and CSE inhibition by approx. 40% and 60%, respectively. This work, while presenting a robust methodological platform for screening putative inhibitors of the human H_2_S-synthesizing enzymes, highlights the importance of employing complementary methodologies in compound screenings.

## Introduction

Once merely regarded as a toxic substance for virtually all life forms, hydrogen sulfide (H_2_S) was discovered to be in fact a ‘gasotransmitter’ in mammalian physiology (similarly to nitric oxide, NO, and carbon monoxide, CO), i.e., an endogenously produced signalling molecule that regulates a myriad of processes like blood flow, cellular stress response, inflammation, apoptosis and energy metabolism^1,2^. H_2_S is endogenously synthesized by specialized enzymes. Yet, it can also be produced by the gut microbiota as an intermediate or end-product of microbial metabolic pathways, or be released from persulfides and polysulfides, endogenously produced or derived from dietary intake (reviewed in^3^). The human enzymes that synthesize H_2_S are cystathionine β-synthase (CBS) and cystathionine γ-lyase (CSE) from the transsulfuration branch of the methionine cycle, and 3-mercaptopyruvate sulfurtransferase (MST)^4^. The signalling function of H_2_S is mostly related to the ability of this molecule to modify target proteins by mediating the persulfidation of cysteine thiols or direct interaction with metal centers, particularly heme moieties (reviewed in^5,6^). Being potentially toxic, H_2_S is disposed via a mitochondrial sulfide oxidation pathway in many cell types^4,7^ and proposedly via haemoglobin in red blood cells^8^. The production and breakdown of H_2_S are kept under tight regulatory control at all levels, transcriptional, translational and post-translational, by complex mechanisms which also involve the other two ‘gasotransmitters’, as reported for CBS^9–14^. Indeed, the relevance of H_2_S homeostasis is attested by the still growing association between altered H_2_S metabolism and human pathologies, from cardiovascular and neurodegenerative diseases to cancer (reviewed in^15,16^). Decreased endogenous H_2_S production, due to CSE knock-out or pharmacological inhibition, leads to hypertension and early development of atherosclerosis (reviewed in^17^). H_2_S has also been implicated in neuronal disorders. Indeed, a correlation between elevated H_2_S levels and amyotrophic lateral sclerosis (ALS) in human patients was reported^18^, while CSE deficiency was found to mediate neurodegeneration in Huntington’s disease^19^. Furthermore, several lines of evidence have shown a close correlation between increased expression of CBS and CSE and different cancer types, namely colorectal, ovarian, breast, prostate and melanoma^20–22^, where increased H_2_S production was suggested to promote cellular proliferation and energy metabolism^22^, and contribute to drug resistance^23,24^. Accordingly, in the human colorectal cancer cell line HCT 116, shRNA-mediated silencing of CBS or its pharmacological inhibition by aminooxyacetic acid (AOAA) significantly impaired cellular proliferation and migration, and tumor xenograft growth^22^.

Given this association between H_2_S metabolism and human disease, several studies have aimed to identify compounds selectively targeting the human H_2_S-synthesizing enzymes^20^. This notwithstanding, currently there are no available potent and selective inhibitors of CBS, CSE and MST suitable for pharmacological applications. AOAA, commonly referred to as a selective CBS inhibitor, also inhibits CSE (and with even higher potency than CBS), as well as several other pyridoxal 5′-phosphate (PLP)-dependent enzymes^25^. Propargylglycine, a well-studied inhibitor of human CSE, though displaying an *IC*_50_ in the tens of micromolar range, requires millimolar concentrations to fully inhibit the enzyme *in vivo*^25^, and only non-selective and weak substrate-like inhibitors have so far been reported for human MST, none of them suitable for biological studies^26^.

Compound screening campaigns have been reported mostly for CBS, but also for CSE and MST. Regarding CBS, screening studies have either focused on direct inhibition of the purified recombinant protein^25,27^, or on cell lines that endogenously overexpress CBS^28,29^. Recently, benserazide was reported to inhibit CBS activity with good selectivity, to impair colon cancer cell proliferation *in vitro*, and tumor growth *in vivo*^29^. As for CSE, a screening has been reported, where a few compounds were observed to inhibit CSE, although they lacked specificity, as they also inhibited CBS^25,30^. Recently, Hanaoka and co-workers performed a compound screening against mouse MST and identified four pyrimidone derivatives with high inhibitory effect against purified MST and MST-overexpressing cells^26^. Despite the potential interest on these newly discovered compounds, there is still the need to discover selective high affinity inhibitors for all three H_2_S-synthesizing enzymes.

High-throughput screening campaigns typically employ orthogonal methods that complement the information from each other and progressively narrow down the number of tested compounds from up to millions of compounds to a few lead molecules^31^. Biophysical methodologies that allow to directly evaluate the interaction between a target protein and the screened compounds are often used as a first line of screening, particularly if it is unfeasible, due to technical and/or budgetary reasons, to employ an enzymatic activity assay in a high-throughput fashion (reviewed e.g. in^32^). First line screening methodologies include differential scanning fluorimetry (DSF, also known as thermal scanning assay), surface plasmon resonance (SPR), nuclear magnetic resonance (NMR), and fluorescence polarization. Once hit compounds are identified in the first line of screening, complementary methodologies, such as *in vitro* activity assays, isothermal calorimetry and X-ray crystallography, ensue to validate lead molecules and feed information into the pipeline for compound optimization. High-throughput H_2_S-detecting methods have thus far commonly employed the fluorescence assay based on the 7-azido-4-methylcoumarin (AzMC) dye. AzMC is selectively reduced by H_2_S to 7-amino-4-methylcoumarin with concomitant increase in fluorescence^33^. Despite its specificity, AzMC not only reacts with H_2_S slowly, but is also prone to chemical interference^29^. Other approaches have been recently reported based on non-commercially available H_2_S probes (e.g.^34^). The colorimetric methylene blue method is one of the most commonly used methods for H_2_S measurement. The method is based on the reaction of H_2_S with *N*,*N*-dimethyl-*p*-phenylenediamine (NNDPD), followed by iron chloride (FeCl_3_)-mediated formation of the methylene blue dye, which is detected by visible absorption spectroscopy^35^.

Herein, we employed a combination of orthogonal biophysical and functional assays to screen a library of synthetic pyridine derivatives against the human H_2_S-synthesizing enzymes CBS, CSE and MST. Both SPR and DSF didn’t detect any strongly interacting compound. An activity-based screening using the H_2_S-detecting AzMC fluorescent probe indicated positive hits. However, a counter-screen with the colorimetric methylene blue method revealed direct interference of the tested compounds with AzMC, thus rebutting the finding of positive hits, and allowed the identification of two compounds weakly inhibiting CBS and CSE. The experimental setup herein presented offers a robust platform for future compound screenings targeting the three human H_2_S-synthesizing enzymes.

## Results

### Synthesis of pyridine derivatives

**Figure 1:**
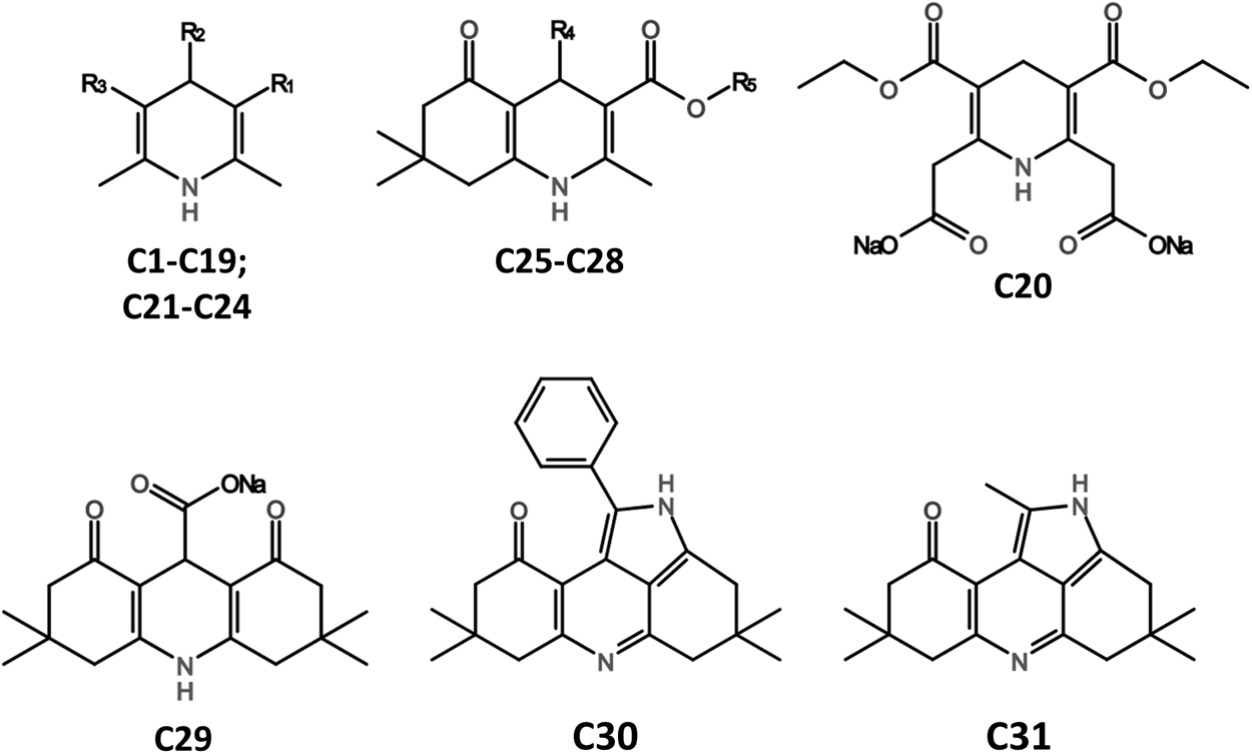
Chemical structures of pyridine derivatives.

Thirty-one pyridine derivatives (Table 1 and Fig. 1) were synthesized and characterized as reported in the Materials and Methods section, employing condensation and heterocyclization reactions. Some of the prepared compounds were used for further transformation to water soluble salts. Compounds purity was at least 95%.

**Table 1:** Pyridine derivatives screened against human H_2_S-synthesizing enzymes.

Comp	R1	R2	R3	R4	R5	Ref.
C1	COOC_2_H_5_	COONa	COOC_2_H_5_	n/a	n/a	^53^
C2	COOCH(CH_3_)_2_	COONa	COOCH(CH_3_)_2_	n/a	n/a	^53^
C3	CN	COONa	CN	n/a	n/a	^53^
C4	COCH_3_	COONa	COCH_3_	n/a	n/a	^53^
C5	COOC_2_H_5_	COOC_2_H_5_	COOC_2_H_5_	n/a	n/a	^53^
C6	COOCH_2_COONa	H	COOCH_2_COONa	n/a	n/a	^54^
C7	COOCH_2_COOC_2_H_5_	H	COOCH_2_COOC_2_H_5_	n/a	n/a	^54^
C8	COOCH_2_COONa	CH_3_	COOCH_2_COONa	n/a	n/a	^54^
C9	COOCH_2_COOC_2_H_5_	CH_3_	COOCH_2_COOC_2_H_5_	n/a	n/a	^54^
C10	COOCH_2_COOC_2_H_5_	C_2_H_5_	COOCH_2_COOC_2_H_5_	n/a	n/a	^54^
C11	COOCH_2_COONa	C_2_H_5_	COOCH2COONa	n/a	n/a	^54^
C12	COOC_2_H_5_	CONHCH(COONa)(CH_2_)_2_COONa	COOC_2_H_5_	n/a	n/a	^51^
C13	COOC_2_H_5_	CONH(CH_2_)_2_SO_3_Na	COOC_2_H_5_	n/a	n/a	^51^
C14	COOC_2_H_5_	CONH(CH_2_)_3_COONa	COOC_2_H_5_	n/a	n/a	^51^
C15	COOC_2_H_5_	CONH(CH_2_)_2_COOH	COOC_2_H_5_	n/a	n/a	^51^
C16	COOC_2_H_5_	COOCH_2_CONH_2_	COOC_2_H_5_	n/a	n/a	This work
C17	COOC_2_H_5_	COOCH_2_COOC_2_H_5_	COOC_2_H_5_	n/a	n/a	This work
C18	COOC_2_H_5_	COOCH_2_COC_6_H_5_	COOC_2_H_5_	n/a	n/a	This work
C19	COOC_2_H_5_	COOCH_2_COC_6_H_4_OCH_3_-4	COOC_2_H_5_	n/a	n/a	This work
C20	n/a	n/a	n/a	n/a	n/a	^56^
C21	COO(CH_2_)_2_COONa	COOCH_3_	COO(CH_2_)_2_COONa	n/a	n/a	^55^
C22	COOCH_2_COOCH_3_	COOH	COOCH_2_COOCH_3_	n/a	n/a	^55^
C23	COOCH_2_COOC_2_H_5_	thienyl	COOCH_2_COOC_2_H_5_	n/a	n/a	^55^
C24	COOCH_2_COONa	thienyl	COOCH_2_COONa	n/a	n/a	^55^
C25	n/a	n/a	n/a	COOH	C_2_H_5_	This work
C26	n/a	n/a	n/a	COOC_2_H_5_	C_2_H_5_	This work
C27	n/a	n/a	n/a	thienyl	CH_2_COOC_2_H_5_	This work
C28	n/a	n/a	n/a	thienyl	CH_2_COONa	This work
C29	n/a	n/a	n/a	n/a	n/a	^52^
C30	n/a	n/a	n/a	n/a	n/a	^50^
C31	n/a	n/a	n/a	n/a	n/a	^50^

### Interaction with human CBS, CSE and MST analyzed by differential scanning fluorimetry (DSF) and surface plasmon resonance (SPR)

**Figure 2:**
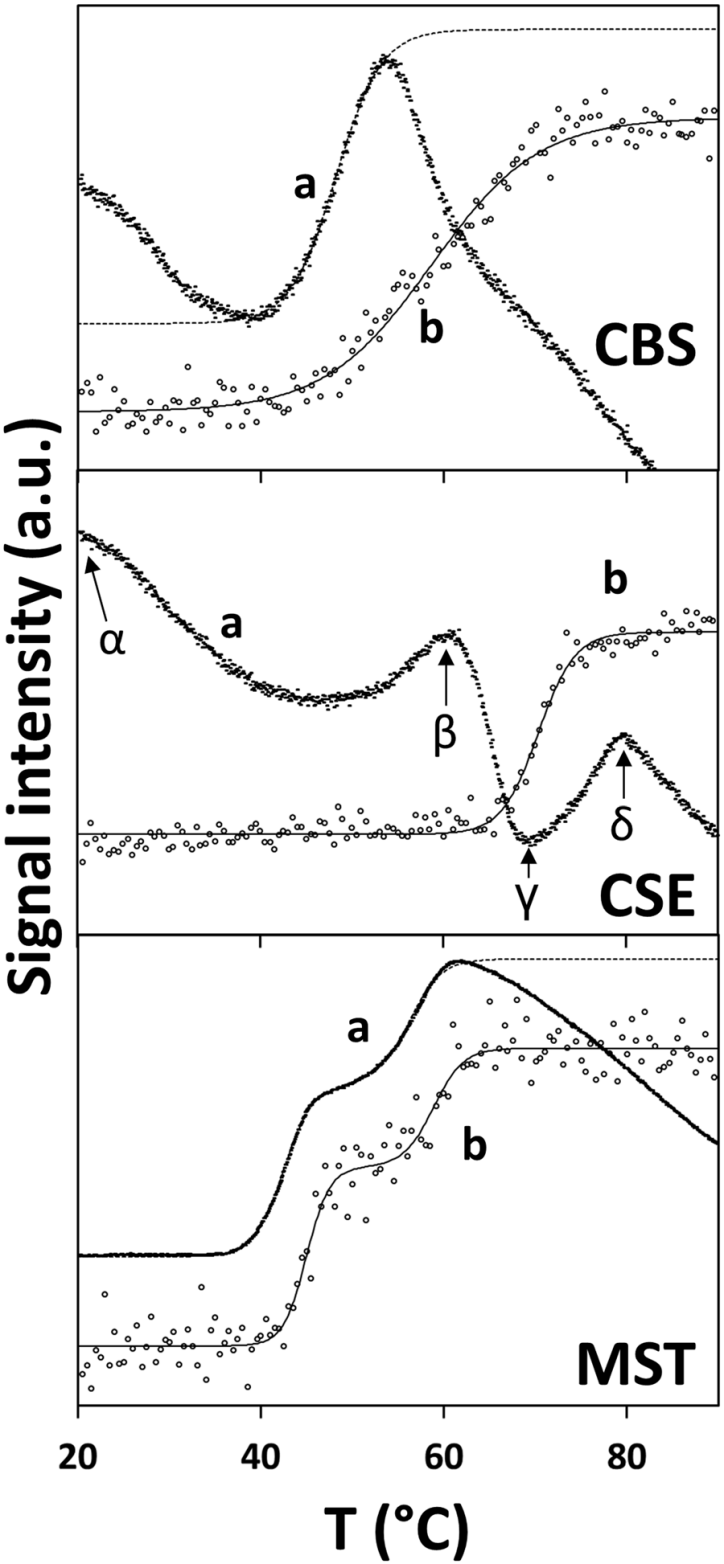
Thermal denaturation profiles of human H_2_S-synthesizing enzymes. Thermal denaturation profiles of recombinant human cystathionine β-synthase (CBS, *top panel*), cystathionine γ-lyase (CSE, *middle panel*) and 3-mercaptopyruvate sulfurtransferase (MST, *bottom panel*), obtained by differential scanning fluorimetry (DSF, short dashed points, curves marked with ‘a’) or Far-UV circular dichroism spectropolarimetry (CD, hollow circles, curves marked with ‘b’).

The newly synthesized derivatives were assayed by two complementary biophysical techniques, namely DSF and SPR, for their ability to bind to the H_2_S-synthesizing human enzymes tCBS, CSE and MST, recombinantly expressed and purified from *E*. *coli*. For each target protein, the DSF assays were preliminarily optimized in terms of protein and dye concentration, resulting in the following conditions (final volume: 20 µL in each well): tCBS (2 µg/well; ~2 μM), CSE (1 µg/well; ~1 μM) or MST (2 µg/well; ~3 μM); final dye concentration: 1x. As shown in Fig. 2 (top panel), the DSF thermal denaturation curve of tCBS (marked with ‘a’) displays an unusually high fluorescence of the dye at the initial temperature (20 °C), indicating either a partial unfolding of the protein or a possible interference from a protein component in the assay. Data were best fitted (line ‘a’ in Fig. 2, top panel) with two consecutive transitions with very close values: *T*_m1_ = 45.7 °C (60%) and *T*_m2_ = 49.8 °C (40%), yielding a weighted mean value *T*_m_‘Ave’_ = 47.4 °C. To check whether tCBS was partly unfolded at the initial temperature of 20 °C, thermal unfolding was also monitored by Far-UV CD spectropolarimetry. As shown in Fig. 2 (top panel), according to the CD thermal denaturation profile acquired at 222 nm (marked with ‘b’), there is no indication of denatured protein at the initial temperature of 20 °C. Moreover, the protein displays an apparent *T*_m_ of 58.6 °C. DSF was employed to screen the effect of the pyridine derivatives at 200 μM concentration on the thermal denaturation profile of tCBS. Statistical validation of the assay was obtained by incubating tCBS with 200 μM AOAA as the negative control (*N* = 25; 6 independent experiments), to be compared with the non-incubated enzyme as positive control (*N* = 23; 6 independent experiments) (Supplementary Fig. S1 and Supplementary Table S1). The assay displayed Z’-factors of −1.58, + 0.08 and −0.28 for *T*_m1,_ *T*_m2_ and *T*_m_‘Ave’_, respectively. Compounds C9, C10, C19 and C23 resulted in aberrant thermal denaturation profiles, precluding any type of analysis. The remaining compounds had a limited impact on the tCBS thermal denaturation (Supplementary Fig. S2). While none of the compounds exhibited |Z-score| ≥ 3.0 for any of the analysed parameters (*T*_m1,_ *T*_m2_ and *T*_m_‘Ave’_), four compounds, namely C2, C14, C28 and C29, yielded |Δ*T*_m_‘Ave’_| ≥ 1.0 °C.

The CSE thermal denaturation profile obtained in the DSF experiments (marked with ‘a’ in Fig. 2, middle panel) presented a basal fluorescence at 20 °C even higher than observed for tCBS. The initial drop preceded two fluorescence increases (at 45–60 °C and 70–80 °C) interspaced by a major drop (at 60–70 °C). By analysing the CSE thermal unfolding monitored by CD spectropolarimetry (marked with ‘b’ in Fig. 2, middle panel), a single transition was detected, with a *T*_m_ at 70.4 °C. While precluding reliable estimates of apparent *T*_m_ values, the DSF thermal denaturation profiles still allow analysing relative changes upon compounds screening, particularly in terms of the high basal fluorescence measured at 20 °C and intensity of each transition. To quantitatively analyse the effect of the pyridine derivatives on CSE thermal denaturation, we established two parameters based on fluorescence ratios along the thermal denaturation profile (marked with Greek letters along the curve in Fig. 2, middle panel, line a): Ratio A = α/β and Ratio B = γ/β. Statistical validation of the assay for CSE was obtained by incubating the enzyme with 200 μM AOAA as the negative control (*N* = 26; 6 independent experiments), to be compared with the non-incubated enzyme as positive control (*N* = 26; 6 independent experiments) (Supplementary Fig. S1 and Supplementary Table S2). The assay displayed Z’-factors of + 0.53 and + 0.31 for Ratios A and B, respectively. Compounds C10, C18, C19 and C23 resulted in aberrant thermal denaturation profiles, precluding any type of analysis. By analysing the effect of the other tested pyridine derivatives on the thermal denaturation profile of CSE (Supplementary Fig. S3), C1, C2, C9, C17, C30 and C31 were identified as the only compounds exhibiting |Z-score| ≥ 3.0 for both Ratio A and Ratio B (Supplementary Table S2).

The MST thermal denaturation profiles obtained by DSF and CD (Fig. 2, bottom panel) exhibited two well separated transitions with nearly matching *T*_m_ values between the two methods: *T*_m1_ = 42.4 °C (60%) and *T*_m2_ = 56.2 °C (40%) by DSF yielding *T*_m_‘Ave’_ = 48.2 °C, to be compared with *T*_m1_ = 45.0 °C (60%) and *T*_m2_ = 58.8 °C (40%) by CD spectropolarimetry yielding *T*_m_ave_ = 50.5 °C. Possible interactions of the pyridine derivatives with MST were evaluated by DSF (Supplementary Fig. S4 and Supplementary Table S3). Statistical validation of the assay for MST was obtained by incubating the enzyme with 3-mercaptopyruvate (3MP, at 2 mM) as the negative control (*N* = 28; 6 independent experiments), to be compared with the non-incubated enzyme as positive control (*N = *31; 6 independent experiments) (Supplementary Fig. S1 and Supplementary Table S3). As shown in Supplementary Fig. S1, the major effect of 3MP was to significantly decrease the relative amplitude of the first transition (‘Frac’ in Supplementary Table S3), while decreasing both *T*_m_ values. The assay displayed Z’-factors of + 0.50, −0.32, + 0.68, and −0.06, for Frac, *T*_m1,_ *T*_m2_ and *T*_m_‘Ave’_, respectively. Compounds C19 and C23 resulted in aberrant thermal denaturation profiles, precluding any type of analysis. Compounds C2, C30 and C31 exhibited |Δ*T*_m1_| ≥ 1.0 °C with |Z-score| ≥ 3.0, while all but six compounds exhibited a |Z-score| ≥ 3.0 with respect to *T*_m2_ (Supplementary Table S3). Five compounds (C5, C13, C26, C27, and C30) resulted in a shift of |Δ*T*_m_‘Ave’_| ≥ 1.0 °C and, notably, out of them only compound C30 exhibited a shift in both *T*_m1_ and *T*_m2_ with |Z-score| ≥ 3.0.

**Figure 3:**
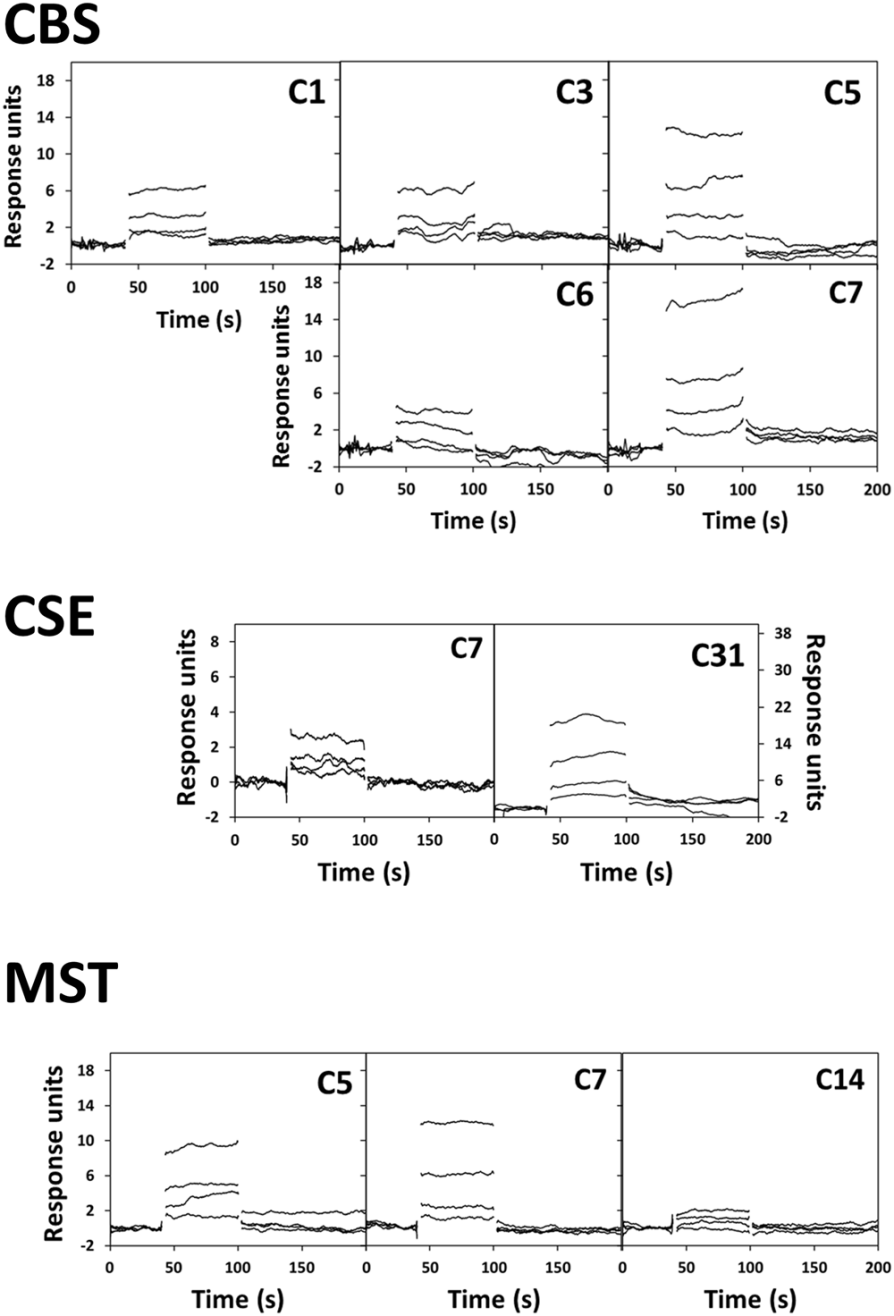
Identification of interacting compounds by surface plasmon resonance. SPR sensorgrams obtained for the interaction between immobilized recombinant human cystathionine β-synthase (CBS, t*op panel*), cystathionine γ-lyase (CSE, *middle panel*) or 3-mercaptopyruvate sulfurtransferase (MST, *bottom panel*) with the indicated pyridine derivatives at 25, 50, 100 and 200 µM.

The synthetic compounds were also screened for their ability to bind CBS, CSE and MST by SPR. This is an optical methodology based on the physical principle of refractive index changes at the biospecific sensor surface upon complex formation^31,36^. After immobilizing the enzymes on the chips, the tested pyridine derivatives were assayed at four concentrations: 25, 50, 100 and 200 µM. No Z’-factor could be measured for these assays due to lack of adequate controls under the selected experimental conditions. Under those conditions, several compounds (C9, C10, C18, C19 and C23) displayed poor solubility, thus precluding an analysis of their interaction with the target proteins by SPR. Moreover, the results obtained for several compounds revealed solubility issues affecting differently the sensorgrams for the three enzymes (Supplementary Figs S5–S7): C1 (MST), C11-C14 (tCBS and CSE), C16 (tCBS and CSE), C17 (all), C20-22 (tCBS and CSE), C24-26 (tCBS and CSE), C27-28 (all), and C31 (tCBS). For the remaining compounds, due to the fast kinetics observed for the studied interactions, no association or dissociation rate constants could be determined. We thus identified compounds yielding sensorgrams where the steady-state response units were proportional to the compound concentration (in the tested range) and did not exceed the expected maximal value (Rmax, based on the compound molecular weight and surface density), thus avoiding any misidentification by superstoichiometric binding behaviour. As shown in Fig. 3, five compounds (C1, C3, C5, C6 and C7) proved to interact with tCBS, two (C7 and C31) with CSE, and three (C5, C7, C14) with MST, in a concentration-dependent mode. The fact that the corresponding steady-state response units linearly increase with the compound concentration up to 200 μM points to low-affinity interactions. Similarly, weak interactions were also observed between AOAA and both tCBS and CSE (Supplementary Fig. S8).

### Inhibition of human CBS, CSE and MST analyzed by fluorimetric and colorimetric assays

**Figure 4:**
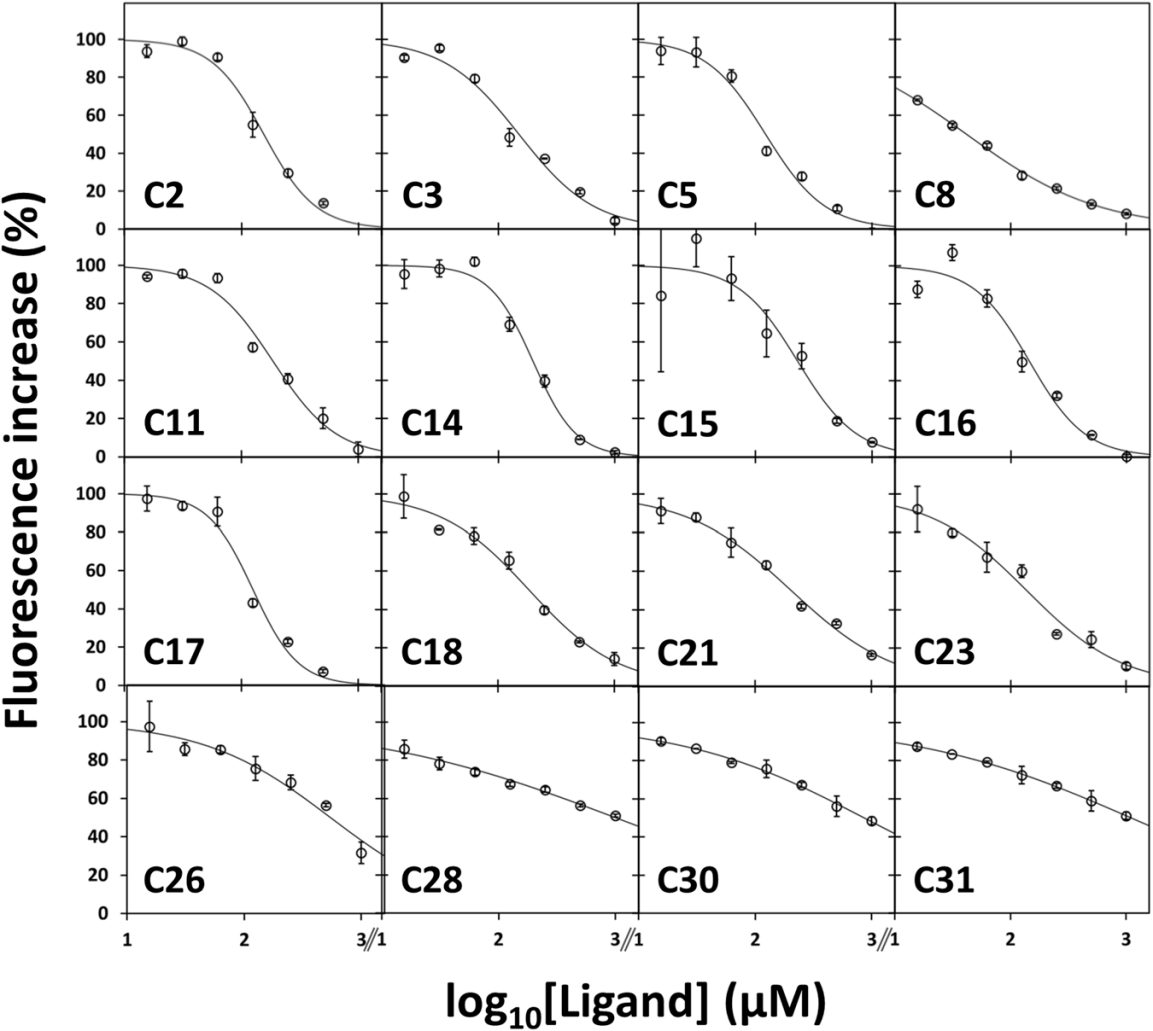
Effect of compounds on fluorimetric H_2_S detection. Effect of the indicated pyridine derivatives on the H_2_S-selective fluorescent probe AzMC. Initial slope of the fluorescence increase on reaction of the probe with H_2_S synthesized by tCBS. Experimental conditions as described in the Materials and Methods section.

The screening was complemented with activity measurements to test the inhibitory efficacy of the synthetic compounds towards the target enzymes. A fluorescence-based assay using the H_2_S-detecting dye AzMC was initially attempted with tCBS alone. A Z’-factor of + 0.78 was obtained for this assay by measuring the fluorescence signal of the dye in the absence of tCBS (negative control; *N* = 16; 3 independent experiments) and in its presence (positive control; *N* = 16; 3 independent experiments) (Supplementary Table S4). All pyridine derivatives were first screened at a single concentration of 200 µM. Under these conditions, sixteen compounds appeared to lower the dye signal (Supplementary Table S4) and were thus assayed in a concentration range between 15.6 µM and 1 mM. As shown in Fig. 4, out of them, twelve seemed rather effective (apparent *EC*_50_ values in the range of 41.6–229.9 µM). However, all the sixteen compounds tested were found to interfere with the AzMc probe, as they markedly decreased the dye signal (see Supplementary Table S5) in control experiments carried out with the H_2_S releaser GYY4137 in place of tCBS, under otherwise identical experimental conditions (Z’-factor = + 0.85, *N* = 16; 3 independent experiments for both positive and negative controls, respectively carried out in the presence and absence of GYY4137). This led us to quit with assays based on the AzMc fluorescent probe and screen the whole compound library on each target enzyme (tCSB, CSE and MST) by detecting H_2_S with the colorimetric methylene blue (MB) assay.

To rule out possible interferences in the MB assay and/or direct reactivity of the compounds with H_2_S, each derivative was tested in preliminary control assays using 3 mM GYY4137 as the H_2_S source (Z’-factor = + 0.84, *N* = 9; 3 independent experiments for both positive and negative controls, respectively carried out in the presence and absence of GYY4137). As shown in Supplementary Table S6, in these assays none of the 31 compounds caused a marked decrease in the colorimetrically detected H_2_S. The MB method was therefore employed to evaluate the effect of each compound on the H_2_S-producing activity of tCBS, CSE and MST (respectively, with Z’-factors = + 0.57, + 0.78 and + 0.51; *N* ≥ 9 in at least 3 independent experiments for both positive and negative controls, respectively carried out in the presence and absence of the proteins, see Table 2). By screening the effect of the whole compound library on the activity of each target enzyme, it was found that, whereas none of the tested derivatives has inhibitory effect towards MST, C30 and C31 are poor inhibitors of both tCBS and CSE (Table 2). Indeed, while 1 mM C30 inhibits tCBS and CSE by approx. 50% and 40%, respectively, 0.5 mM C31 accounts for tCBS and CSE inhibition by approx. 40% and 60%, respectively.

**Table 2:** Effect of pyridine derivatives on the H_2_S-synthesizing activity of tCBS, CSE and MST.

	tCBS			CSE			MST		
	H_2_S production (%) (Z’ factor: 0.60)	CV	Z-score	H_2_S production (%) (Z’ factor: 0.78)	CV	Z-score	H_2_S production (%) (Z’ factor: 0.51)	CV	Z-score
Control	100.0 (N = 18)	11.6	—	100.0 (N = 9)	12.0	—	100.0 (N = 9)	6.8	
Blank	5.4 (N = 18)	37.2	8.2	5.2 (N = 9)	27.5	7.9	16.9 (N = 9)	48.5	12.1
**AOAA**	8.8	12.1	**7.9**	6.7	59.7	**7.8**	—	—	—
C1*	98.4	3.4	0.1	102.3	2.8	−0.2	103.0	7.8	−0.4
C2	92.4	4.2	0.7	98.4	2.7	0.1	102.5	0.5	−0.4
C3	89.5	5.7	0.9	96.7	2.5	0.3	103.9	5.3	−0.6
C4	77.4	8.2	2.0	101.0	3.0	−0.1	100.0	4.1	0.0
C5	93.5	7.9	0.6	122.0	0.0	−1.8	107.5	0.6	−1.1
C6*	79.8	6.6	1.7	92.6	0.9	0.6	99.7	4.0	0.0
C7	95.1	2.1	0.4	97.3	1.8	0.2	99.9	6.8	0.0
C8	92.4	9.8	0.7	94.0	1.4	0.5	87.4	7.5	1.8
C9	97.1	1.7	0.2	90.3	3.6	0.8	96.5	3.4	0.5
C10	103.8	11.9	−0.3	98.0	1.0	0.2	98.0	3.7	0.3
C11	108.6	7.3	−0.7	106.9	8.4	−0.6	89.6	0.5	1.5
C12*	97.0	16.7	0.3	90.8	5.4	0.8	103.6	4.0	−0.5
C13	97.9	8.7	0.2	89.8	2.4	0.9	93.1	3.7	1.0
C14	85.0	2.8	1.3	93.0	6.5	0.6	106.3	7.4	−0.9
C15	80.7	1.1	1.7	92.6	2.4	0.6	91.2	2.1	1.3
C16	122.5	1.7	−2.0	93.8	15.3	0.5	104.8	7.9	−0.7
C17	109.2	3.9	−0.8	97.2	7.6	0.2	102.3	3.9	−0.3
C18	105.2	13.2	−0.5	94.4	2.3	0.5	101.9	2.1	−0.3
C19	118.4	3.2	−1.6	96.6	4.4	0.3	103.9	6.6	−0.6
C20*	101.5	1.3	−0.1	116.2	18.8	−1.4	103.4	9.0	−0.5
C21*	79.0	7.6	1.8	101.0	1.0	−0.1	103.3	3.1	−0.5
C22	68.3	4.2	2.7	100.6	8.2	−0.1	107.7	1.8	−1.1
C23	115.8	8.7	−1.4	91.3	3.3	0.7	103.4	2.0	−0.5
C24*	94.7	5.5	0.5	112.2	1.6	−1.0	101.6	3.6	−0.2
C25	94.7	14.1	0.5	102.9	21.7	−0.2	106.6	3.5	−1.0
C26	97.5	23.2	0.2	99.8	1.5	0.0	97.2	4.0	0.4
C27	104.8	4.9	−0.4	96.2	3.6	0.3	98.8	1.3	0.2
C28	93.8	16.7	0.5	91.8	14.0	0.7	108.0	2.7	−1.2
C29	81.3	22.5	1.6	97.4	5.3	0.2	107.6	6.4	−1.1
**C30**	50.3	12.9	**4.3**	64.2	9.7	**3.0**	130.3	2.3	−4.4
C31*	60.7	24.6	**3.4**	38.4	5.1	**5.1**	119.1	6.8	−2.8

*Compounds tested at a final concentration of 500 µM rather than 1 mM.

## Discussion

Hydrogen sulfide (H_2_S) is endogenously produced to accomplish the regulation of numerous physiological processes, ranging from neoangiogenesis^37^, vasorelaxation and blood pressure^38^ to cardioprotective^39^, antinflammatory^40^ and antioxidant^41^ effects. Altered H_2_S metabolism is associated with multiple human pathologies, such as cardiovascular^17^ and inflammatory^42^ disorders, neurodegeneration^19^ and cancer^22,43^. Therefore, the development of compounds targeting the three H_2_S-synthesizing enzymes, namely CBS, CSE and MST, may prove beneficial for future therapeutic strategies, as posited for CBS^20,28^. To date, only unspecific or relatively weak inhibitors have been reported for CBS. While AOAA displays a half-maximal inhibitory concentration (*IC*_50_) of approximately 3 μM for human recombinant CBS, it also inhibits human CSE with even higher potency, as well as other PLP-dependent enzymes^25^. Whereas benserazide appears more specific towards CBS, it exhibits lower potency^29^. The present work attempted to identify new CBS, CSE and MST inhibitor scaffolds, using combined biophysical and biochemical approaches. The tested compounds were assembled in a composite library of 31 pyridine derivatives.

A combination of orthogonal biophysical and biochemical methods was applied to provide a robust and effective platform to identify putative ligands. Despite the fact that both DSF and SPR are able to detect protein-ligand interactions, though through different mechanisms, frequently in compound screenings the lists of hits identified by either method are only partially overlapping. Both methodologies are typically employed due to the ease of setting up such assays, and to the relatively low amounts of protein required (reviewed e.g. in^32,44,45^). The usage of both methodologies thus affords a more robust approach^46^.

Herein, DSF data required a rather elaborate analysis due to different factors. The DSF denaturation profiles of tCBS presented a relatively high basal fluorescence (unrelated to protein destabilization at the initial temperature as judged by the CD-monitored thermal denaturation profile) and were best fitted with two apparent *T*_m_ values (Fig. 2). The discrepancy between the CD- and DSF-monitored thermal denaturation profiles and corresponding *T*_m_ values could be due to interference of the tCBS cofactors, through fluorescence increase and/or quenching by PLP and heme iron, respectively. Regardless of cofactor interference, DSF could still be employed to evaluate the effect produced by each compound on the protein thermal denaturation profile and four compounds (C2, C14, C28 and C29) were found to mildly affect tCBS thermal stability (|Δ*T*_m_‘Ave’_| ≥ 1.0 °C), though with |Z-score| < 3.0 (Supplementary Table S1).

Similarly to tCBS, CSE showed high basal fluorescence intensity at resting temperature, possibly related to the presence of the PLP cofactor. Indeed, the CD-monitored thermal denaturation profile revealed CSE to be remarkably stable, ruling out protein instability at resting temperature (Fig. 2). Despite the unconventional DSF thermal denaturation profile, it was still employed to survey possible interactions of the pyridine derivatives with CSE. Based on two parameters (Ratio A and B) quantitatively evaluating profile shape changes, both statistically validated by using the CSE inhibitor AOAA as negative control, six compounds (C1, C2, C9, C17, C30 and C31) were identified as putative interactors for CSE (Supplementary Table S2). These compounds indeed proved to be the only ones causing changes in both parameters with |Z-score| ≥ 3.0.

MST revealed a better agreement between the DSF and CD thermal denaturation profiles, with two well defined transitions likely associated with the two homologous domains that compose MST^47^ and other transulfurases, like rhodanese^48^. From the four analysed parameters (Frac, *T*_m1_, *T*_m2_, *T*_m_‘Ave’_), compounds putatively interacting with MST were identified based on a combination of defined criteria related to *T*_m_ shifts and corresponding Z-score values. According to these criteria, only two compounds (C2 and C30) were found to cause shifts in both *T*_m1_ and *T*_m2_ with |Z-score| ≥ 3.0, and notably C30 caused the greatest Δ*T*_m_‘Ave’_ (−1.4 °C) among all tested compounds (Supplementary Table S3).

Despite the limited identification of strong hits within the tested compound library, DSF assays under the experimental conditions used in this study proved to be suitable to screen compounds, at least for CSE and MST, while further optimization of the assay seems to be required in the case of tCBS, particularly for high-throughput screenings. Indeed, for the assay on CSE, according to^49^, Ratio A exhibited a Z’-factor (+0.53) consistent with an excellent assay, while Ratio B exhibited a slightly lower Z’-factor (+0.31), yet consistent with a good assay (Supplementary Table S1). Likewise, for the assay on MST, two parameters (Frac and *T*_m2_) were found to be characterized by Z’-factors (+0.50 and +0.68, respectively) consistent with an excellent assay (Supplementary Table S2). In contrast, for the assay on tCBS, only *T*_m2_ exhibited a slightly positive Z’-factor (+0.08), unlike *T*_m1_ and *T*_m_‘Ave’_ (displaying Z’-factor values of −1.58 and −0.28) (Supplementary Table S3).

In parallel with the DSF assays, putative interactions of the tested compounds with the three H_2_S-synthesizing enzymes were investigated by surface plasmon resonance (SPR). The disadvantages of using a fluorescence-based technique involving an added dye are overcome by SPR, which is a label-free methodology. Herein a CM5 chip was employed to immobilize all target proteins, based on covalent linkage to the carboxymethylated surface of exposed lysine residues distributed through the protein surface, thus offering different possible binding orientations. Pyridine derivatives were screened at four concentrations, from 25 to 200 μM, which already proved to be above the desired solubility for 15 compounds (approximately half of this library). Despite this limitation and the current lack of adequate controls to statistically validate the use of SPR for high-throughput screening, we observed interactions for a limited number of compounds, based on the proportionality between steady-state response units and compound concentration: 15% of the compounds in the library for tCBS, ~6% for CSE and ~10% for MST (Fig. 3). For these compounds, however, it was not possible to extrapolate binding affinities, as the steady-state response units linearly increased with the compound concentration pointing to low-affinity interactions. The results herein obtained for the pyridine derivatives suggest that the use of SPR in future compound screenings targeting CBS, CSE and MST would require further optimization and proper statistical validation.

The 31 pyridine derivatives were then assayed for their ability to inhibit H_2_S production by tCBS using the H_2_S-selective fluorescent probe AzMC^29^. While 16 compounds were selected as ‘positive hits’ based on a detailed kinetic analysis evaluating these compounds in a wide concentration range (Fig. 4), control experiments were performed replacing tCBS with the H_2_S donor GYY4137, revealing that all the tested compounds strongly interfered with AzMc detection of GYY4137-generated H_2_S (Supplementary Table S5 and Supplementary Fig. S9). This apparent drawback, similar to the interference of reference CBS inhibitor NSC67078^27^ with the AzMc probe reported by Druzhyna and co-workers^29^, further highlights the caveat of fluorescence-based methods, particularly when dealing with compounds and/or fragments with aromatic moieties. The discovery that the presumed 16 ‘hit’ pyridine derivatives interfered with the AzMc fluorescence-based method prompted us to employ the MB method in 96-well plates as described in^29^, adapted to prevent H_2_S loss upon addition of zinc acetate. After confirming that none of the 31 compounds interfered with MB detection of H_2_S released by GYY4137 (Supplementary Table S6), the compounds were screened against the three target proteins. Whereas none of the pyridine derivatives affected MST (Table 2), compounds C30 and C31 appeared to partially inhibit both CBS and CSE (Table 2), albeit only at relatively high concentrations (respectively, 1 and 0.5 mM). Interestingly, C30 and C31 share the same molecular scaffold (Fig. 1). It is worth noting that all fluorimetric and colorimetric assays herein described under the experimental conditions used in the present study proved to be suitable even for high-throughput screenings based on the determined Z’-factor values^49^ (Table 2 and Supplemental Tables S4–S6).

Herein, orthogonal biophysical screening methods, based both on protein-compound interaction (DSF and SPR) and on enzymatic inhibition (H_2_S-detection assays), were employed for the first time for the three human H_2_S-synthesizing enzymes CBS, CSE and MST altogether. Testing a library of 31 pyridine derivatives, a relatively low overlap was observed between DSF and SPR outputs in terms of compounds possibly interacting with the target enzymes. The possibility of including functional approaches such as enzymatic inhibition assays in screening campaigns increases the robustness of the resulting hit compound identification. Herein, only two out of the 31 tested compounds, namely C30 and C31, displayed a weak inhibitory activity towards both tCBS and CSE. Notably, compound C31 was also identified by DSF as a hit compound for CSE and likely interactor of the same protein by SPR.

In conclusion, the drug discovery process can be viewed as a bumpy ride where methodological limitations may lead to the identification of false positives as hit molecules. In this regard, the present study further highlights the importance in compound screening campaigns of crossing the read-outs from complementary methodological approaches, ranging from the investigation of biophysical dynamic aspects (such as protein-ligand binding) to the implementation of more functional assays (enzymatic activity). From this perspective, we hope that the experimental setup herein presented, that integrates analysis of protein-ligand interactions by both DSF and SPR, and protein thermal denaturation investigation by Far-UV CD, with activity assays based on fluorimetric and colorimetric H_2_S detection methods, offers a robust platform for the discovery of hit compounds to develop selective and potent inhibitors of the three human H_2_S-synthesizing enzymes.

## Methods

### Chemicals

All chemicals were purchased from Sigma, except GYY4137 (from CAYMAN), the Protein Thermal Shift Dye Kit™ (from Applied Biosystems) and the SPR Amine Coupling Kit, type 2 (from GE Healthcare).

### Synthesis and characterization of pyridine derivative compounds

Herein, a composite library of thirty-one pyridine derivatives was assembled (Table 1 and Fig. 1), based on newly synthesized (C16-C19 and C25-C28) and previously reported compounds (C1-15, C20-24 and C29-C31)^50–56^. Compound purity was assessed by high-performance liquid chromatography in a Waters Alliance system coupled to a Waters 2485 UV/Vis detector.

#### 2,6-Dimethyl-1,4-dihydropyridine-3,4,5-tricarboxylic acid 4-carbamoylmethylester 3,5-diethylester (C16)

To the mixture of 0.3 g (1 mmol) 2,6-dimethyl-1,4-dihdropyridine-3,4,5-tricarboxylic acid 3,5-diethyl ester in 5 ml of dimethylformamide, 0.37 g (2 mmol) potassium carbonate and 0.19 g (1 mmol) iodoacetamide were added. The reaction mixture was heated at 60 °C for 3 h. Afterwards, the reaction mixture was evaporated, and the residue was dissolved in water and extracted with dichloromethane. The dichloromethane layer was dried over magnesium sulfate, filtered and evaporated. Residue was crystallized with diethyl ether and 0.2 g (56.5%) of compound C16 was obtained.

Compound C16: ^1^H-NMR (400 MHz, DMSO-d_6_): 8.98 (s, 1 H), 7.36 (s, 1 H), 6.85 (s, 1 H), 4.71 (s, 1 H), 4.32 (s, 2 H), 4.16–4.02 (m, 4 H), 2.23 (s, 6 H), 1.19 ppm (t, *J* = 7.04 Hz, 6 H).

m/z = 353[M-H]^−^. Mp. = 183–184 °C.

Calculated for C_16_H_22_N_2_O_7_: C, 54.23; H, 6.26; N, 7.91. Found: C, 54.25; H, 6.22; N, 7.80.

#### 2,6-Dimethyl-1,4-dihydropyridine-3,4,5-tricarboxylic acid 4-ethoxycarbonylmethyl ester 3,5-diethylester (C17)

To the mixture of 1 g (3.3 mmol) 2,6-dimethyl-1,4-dihdropyridine-3,4,5-tricarboxylic acid in 35 ml acetone, 1.36 g (9.9 mmol) potassium carbonate was added. The obtained mixture was stirred and refluxed, and 0.56 g (3.3 mmol) of bromo-acetic acid ethyl ester was added. Then, the reaction mixture was refluxed for 4 h, cooled to room temperature and the formed potassium bromide was filtered off. The filtrate was evaporated, dissolved in 15 ml of ethanol and left for 12 h in the refrigerator for crystallization. After filtration and crystallization of the obtained precipitate from benzene, 0.78 g (61%) of compound C17 was obtained.

Compound C17: ^1^H-NMR (400 MHz, CDCl_3_): 5.84 (s, 1 H), 4.98 (s, 1 H), 4.53 (s, 2 H), 4.25–4.12 (m, 6 H), 2.32 (s, 6 H), 1.29–1.21 ppm (m, 9 H).

m/z = 382[M-H]^−^. Mp. = 110–111 °C.

Calculated for C_18_H_25_NO_8_: C, 56.39; H, 6.57; N, 3.65. Found: C, 56.54; H, 6.55: N, 3.52.

#### 2,6-Dimethyl-1,4-dihydropyridine-3,4,5-tricarboxylic acid 3,5-diethylester 4-(2-oxo-2-phenylethyl)ester (C18)

Compound C18 (58% yield) was prepared similarly to C17.

Compound C18: ^1^H-NMR (400 MHz, CDCl_3_): 7.86–7.42 (m, 5 H), 6.19 (s, 1 H), 5.23 (s, 2 H), 5.05 (s, 1 H), 4.26–4.14 (m, 4 H), 2.30 (s, 6 H), 1.27 ppm (t, *J* = 7.04 Hz, 6 H).

m/z = 414[M-H]^−^. Mp. = 126–128 °C.

Calculated for C_22_H_25_NO_7_: C, 63.61; H, 6.7; N, 3.33. Found: C, 63.70; H, 6.03; N, 3.32.

#### 2,6-Dimethyl-1,4-dihydropyridine-3,4,5-tricarboxylic acid-3,5-diethylester-4-[2-(4-methoxy-phenyl)−2-oxo-ethyl] ester (C19)

Compound C19 (57% yield) was prepared similarly to C17.

Compound C19: ^1^H-NMR (400 MHz, DMSO-d_6_): 8.96 (s, 1 H), 7.87 (d, *J* = 4 Hz, 2 H), 7.03 (d, *J* = 4 Hz, 2 H), 5.26 (s, 2 H), 4.84 (s,1 H), 4.16–4.03 (m, 4 H), 3.84 (s, 3 H), 2.24 (s, 6 H), 1.19 ppm (t, *J* = 7.04 Hz, 6 H).

m/z = 444[M-H]^−^. Mp. = 155–156 °C.

Calculated for C_23_H_27_NO_8_: C, 62.01; H, 6.11; N, 3.14. Found: C, 61.72; H, 6.02; N, 3.04.

#### 2,7,7-Trimethyl-5-oxo-1,4,5,6,7,8-hexahydroquinoline-3,4-dicarboxylic acid 3-ethyl ester (C25)

Sodium hydroxide (0.5 g, 12.5 mmol) in 3 ml of water was added to a solution of 2 g (6 mmol) ester C26 in 10 ml of ethanol and mixed for 40 min. Then, the reaction mixture was evaporated and the residue dissolved in water. The water solution was acidified to pH = 5 with hydrochloric acid, and the obtained precipitate was filtered and crystallized from ethanol. 1.06 g (58%) of compound C25 was obtained.

Compound C25: ^1^H-NMR (400 MHz, DMSO-d_6_): 11.79 (s, 1 H), 9.10 (s, 1 H), 4.52 (s, 1 H), 4.13–4.00 (m, 2 H), 2.35 (dd, *J* = 17.2 Hz, 2 H), 2.22 (s, 3 H), 2.16 (dd, *J* = 17.2 Hz, 2 H), 1.18 (t, *J* = 7.0 Hz, 3 H), 1.02 (s, 3 H), 1.00 ppm (s, 3 H).

m/z = 308[M + H]^+^. Mp. = 165–170 °C.

Calculated for C_16_H_21_NO_5_^.^ H_2_O: C, 59.07; H, 7.13; N, 4.30. Found: C, 59.58; H, 7.07; N, 4.26.

#### 2,7,7-Trimethyl-5-oxo-1,4,5,6,7,8-hexahydroquinoline-3,4-dicarboxylic acid diethyl ester (C26)

To 13 g (0.1 mol) of ethylacetoacetate in 0.2 ml of piperidine, 0.2 ml acetic acid and 20.4 ml (0.1 mol) of a 50% solution of ethyl glyoxalate in toluene was added. The obtained solution was mixed for 48 h at room temperature. Then, 13.9 g (0.1 mol) of dimedone enamine in 30 ml of ethanol was added to the reaction mixture and refluxed for 1 h. After cooling, the mixture began to form precipitates which were filtered and crystallized from ethanol and as a result 10 g (30%) of compound C26 was obtained.

Compound C26: ^1^H-NMR (400 MHz,CDCl_3_): 5.95 (s, 1 H), 4.85 (s, 1 H), 4.23–4.01 (m, 4 H), 2.33 (dd, *J* = 17.2 Hz, 2 H), 2.30 (s, 3 H), 2.27 (dd, *J* = 16.4 Hz, 2 H), 1.18 (t, *J* = 7.0 Hz, 3 H), 1.11 (s, 3 H), 1.09 ppm (s, 3 H).

m/z = 336[M + H]^+^. Mp. = 194–196 °C.

Calculated for C_18_H_25_NO_5_: C, 64.46; H, 7.51; N, 4.18. Found: C, 64.41; H, 7.41; N, 4.12.

#### 2,7,7-Trimethyl-5-oxo-4-thiophen-2-yl-1,4,5,6,7,8-hexahydroquinoline 3-carboxylic acid ethoxycarbonylmethyl ester (C27)

A mixture of 0.7 g (5 mmol) of 3-amino-5,5-dimethyl-cyclohex-2-enone, 0.56 g (5 mmol) of 2-thiophene carboxaldehyde and 0.95 g (5 mmol) of 3-oxo-butyric acid ethoxycarbonylmethyl ester in 10 ml of ethanol was stirred at room temperature for 3 days. The reaction mixture was cooled to −10 °C and the product crystallization was observed. The crude product was recrystallized from ethanol and 0.85 g (42%) of C27 was obtained.

Compound C27: ^1^H-NMR (400 MHz, DMSO-d_6_): 9.36 (s, 1 H), 7.17 (m, 1 H), 6.81 (m, 1 H), 6.72 (m, 1 H), 5.20 (s, 1 H), 4.65 (d, *J* = 16 Hz, 2 H), 4.10 (kv, *J* = 7.0 Hz, 2 H), 2.37 (dd, *J* = 17.2 Hz, 2 H), 2.27 (s, 3 H), 2.15 (dd, *J* = 16.0 Hz, 2 H), 1.17 (t, *J* = 7.0 Hz, 3 H), 1.02 (s, 3 H), 0.93 ppm (s, 3 H).

m/z = 404[M + H]^+^. Mp. = 188–190 °C.

Calculated for C_21_H_25_NO_5_S: C, 62.51; H, 6.25; N, 3.47. Found: C, 62.41; H, 6.34; N, 3.34.

#### 2,7,7-Trimethyl-5-oxo-4-thiophen-2-yl-1,4,5,6,7,8-hexahydroquinoline 3-carboxylic acid carboxymethyl sodium salt (C28)

To a hot solution of 0.3 g (0.74 mmol) of methylester C27 in 7 ml of ethanol, a solution containing 0.033 g (0.83 mmol) of sodium hydroxide in 0.8 ml of water was added and boiled for 3 minutes. The solvent was evaporated. The obtained precipitates of crude product were suspended in diethyl ether. After filtration and washing with diethyl ether, 0.26 g (90%) of sodium salt C28 was obtained.

Compound C28: ^1^H-NMR (400 MHz, DMSO-d_6_): 7.11 (m, 1 H), 6.79–6.76 (m, 2 H), 5.21 (s, 1 H), 4.15 (dd, *J* = 14.4 Hz, 2 H), 2.35 (dd, *J* = 16.0 Hz, 2 H), 2.27 (s, 3 H), 2.15 (dd, *J* = 16.0 Hz, 2 H), 1.01 (s, 3 H), 0.94 ppm (s, 3 H).

m/z = 374[M-Na + H]^−^. Mp. = 195–196 °C.

Calculated for C_19_H_20_NNaO_5_S·2,5H_2_O: C, 51.57; H, 5.65; N, 3.16. Found: C, 51.24; H, 5.10; N, 3.03.

### Protein expression and purification

A truncated version of human CBS (tCBS), lacking the *s*-adenosyl-l-methionine-binding regulatory domain, was expressed and purified as described in^12^. Human CSE and MST were expressed and purified as described in Sun *et al*.^30^ and in Yadav *et al*.^47^ respectively.

### Differential scanning fluorimetry (DSF)

DSF allows to gain information on ligand binding to a target protein from the observed changes in the protein thermal denaturation profile^57^. Using 96-to-1536-well plates in an RT-PCR instrument, protein denaturation is monitored as a function of temperature increase by making use of a fluorescent dye that emits light upon binding to the buried hydrophobic amino acid residues that become exposed as the protein unfolds. Typically, thermal denaturation profiles are sigmoidal and the protein melting temperature (*T*_m_) is estimated from the inflexion point. Ligand binding is evaluated from the ability of a given compound to either stabilize or destabilize the target protein, which is reflected in an increase or decrease in the *T*_m_. Here, DSF measurements were carried out in 384-well plates in an Applied Biosystems QuantStudio™ 7 Flex Real-Time PCR. Prior to testing the compounds, the assay was optimized for each target protein in terms of concentration of the proteins (1 to 6 μM) and the fluorescent dye (1x, 2x and 4x; the Protein Thermal Shift Dye from Applied Biosystems is commercially available in a 1000 x concentration, without disclosing the actual molar concentration). Once conditions were optimized (see Results), the compounds were assayed by mixing in each well 14 µl of tCBS (2 μg/well; ~2 μM), CSE (1 μg/well; ~1 μM), or MST (2 μg/well; ~3 μM) in Buffer A (10 mM HEPES, 150 mM NaCl, 1 mM tris(2-carboxyethyl)phosphine [TCEP], pH 7.2), with 2 µl of compound solution (typically 2 mM in 10% dimethyl sulfoxide [DMSO], yielding a final compound concentration of 200 µM). The microplate was incubated at 4 °C for 2 hours to favour possible interactions with the target proteins. Prior to the measurement, the fluorescent dye was pre-diluted in Buffer A to a 5x working concentration, and 4 µl of the dye solution were dispensed into each well. The microplate was sealed with optical adhesive covers (Applied Biosystems) and centrifuged for 30 s at 500 × *g*. Using the QuantStudio™ 7 Flex Real-Time PCR System, the microplates were incubated at increasing temperatures, from 20 °C to 90 °C with increments of 1 °C/min. Protein unfolding was monitored with the ROX filter (excitation wavelength = 580 ± 10 nm and emission wavelength = 623 ± 14 nm). Data from triplicate curves were averaged and, for tCBS and MST, the melting temperatures were determined by fitting the data to biphasic sigmoidal curves (for CSE, please see the Results section). For statistical validation of the DSF assays towards these protein targets, control assays were performed employing 200 μM AOAA as negative control both for tCBS and CSE, and 2 mM 3-mercaptopyruvate for MST, with the compound-free enzymes being used as positive controls.

### Far-UV circular dichroism

Far-UV circular dichroism (CD) thermal denaturation curves were recorded in a Jasco J-815 spectropolarimeter equipped with a Jasco CDF-426S Peltier temperature controller, in a 0.1 cm path length quartz cuvette. CSE was diluted to 0.15 mg/mL in 50 mM NaPi buffer, 300 mM NaCl, 0.5 mM TCEP, 10% glycerol, pH 7.5, while tCBS and MST were diluted to 0.2 mg/mL in 10 mM Hepes buffer, 150 mM NaCl, 1 mM TCEP, pH 7.2. CD spectropolarimeter experimental setup: λ = 222 nm; temperature range, 20–90 °C; slope, 1 °C/min; data pitch, 0.5 min; data integration time, 1 s; bandwidth, 1 nm; nitrogen flow, 4 L/min. Thermal denaturation curves were analyzed with Graphpad (Prism), fitting the data to a monophasic (tCBS and CSE) or biphasic (MST) sigmoidal curve.

### Surface plasmon resonance (SPR)

Putative interactions of the tested compounds with tCBS, CSE, and MST were investigated by surface plasmon resonance (SPR). Assays were carried out in a Biacore 4000 instrument (GE Healthcare) at 25 °C. First, a pH scouting was performed on a CM5 chip for immobilization optimization using the following buffers: 10 mM sodium acetate pH 5.0, 5.5 and 5.8, 10 mM Bis-Tris pH 6.0 and 6.5, 10 mM sodium phosphate pH 7.0, 10 mM HEPES pH 7.5, or 10 mM Tris-HCl pH 7.5.

tCBS, CSE and MST were diluted to 5 µg/mL (tCBS) or 10 µg/mL (CSE and MST) in their corresponding immobilization buffer (10 mM sodium acetate pH 5.8 for CBS, and 10 mM Bis-Tris pH 6.0 for CSE and MST) and immobilized onto CM5 sensor chips using the standard amine coupling procedure. HBS-N, which consisted of 10 mM Hepes pH 7.4 and 150 mM NaCl, was used as the background buffer. Prior to immobilization, the carboxymethylated surface of the chip was activated with 20 mM 1-ethyl-3-(3-dimethylaminopropyl)-carbodiimide and 5 mM *N*-hydroxysuccinimide for 1.5 min. Proteins were coupled to the surface with a 5 to 10 min injection time at a flow rate of 10 µL min^−1^ in order to reach 5000 to 10000 response units (RU). The remaining activated carboxymethylated groups were blocked with a 5 min injection of 1 M ethanolamine pH 8.5.

Compounds (analytes) were directly diluted in running buffer (10 mM HEPES, 150 mM NaCl, 1 mM TCEP, 0.1 mM EDTA, 0.05% (v/v) TWEEN-20, 5 mM MgCl_2_, pH 7.2.) and injected at four different concentrations using 2-fold dilutions series, with the highest concentration tested being 200 µM. Interactions were qualitatively assessed from the obtained plots of steady-state analyte binding levels against the concentration, making use of the provided Biacore 4000 evaluation software (GE Healthcare).

### Fluorimetric H_2_S-producing activity assays

Fluorimetric H_2_S production activity assays were adapted from Thorson *et al*.^33^. Assays were carried out in 96-well black plates, using the H_2_S-selective fluorescent probe AzMC and a FLUOstar Optima BMG Labtech plate reader. The reaction mixture, in 200 mM Tris-HCl pH 8.0, contained 1.12 µg recombinant human tCBS per well (100 nM), 0.5 mM homocysteine, 50 µM PLP, and 50 µM AzMc (diluted from a 49.7 mM stock in DMSO). Compounds dissolved in DMSO were added to each well to yield a final concentration ranging from 15.6 µM to 1 mM (5% DMSO) in a total assay volume of 250 µl. The plate was incubated at 37 °C for 10 min and the reaction was then triggered by adding the 10 mM l-cysteine. The increase in the probe fluorescence (λ_excitation_ = 340 nm; λ_emission_ = 460 nm) was monitored over 1 hour at 37 °C. The reader was set up to automatically shake the plate for 5 seconds prior to each data acquisition. Data were analysed using Excel and activity was calculated from the initial slope of the fluorescence increase after l-cysteine addition. Control experiments to evaluate a possible interference of the tested compounds with the probe were done by replacing tCBS with the H_2_S donor GYY4137 (3 mM in DMSO, final concentration).

### Counterscreen using the methylene blue assay

The colorimetric methylene blue assay, commonly used to detect H_2_S, was adapted to 96-well plates, as reported by Druzhyna *et al*. for CBS^29^, and improved in order to i) avoid H_2_S escape from the reaction mixture and headspace before trapping sulfide with zinc acetate, and ii) be extended to the other two H_2_S-synthesizing enzymes (CSE and MST). Prior to the enzymatic inhibition assays, control absorbance measurements were performed in 96-well plates: at λ = 600 nm to evaluate the compounds solubility under the assays conditions, and at λ = 690 nm to evaluate their interference with the methylene blue read outs (and employ a correction factor whenever needed).

For CBS- and CSE-catalyzed H_2_S production assays, 110 µL of reaction mixture contained 0.5 mM or 1 mM of tested compound, tCBS (10 µg/well) or CSE (30 µg/well) in 50 mM Tris-HCl pH 8.0, and 5 µM PLP. In the case of tCBS, 2 mM homocysteine was also added. After keeping the plate on ice for 30 min, the reactions by tCBS and CSE were respectively triggered by adding either l-cysteine (10 mM final concentration) alone or plus homocysteine (2 mM final concentration), The plate was sealed with vinyl adhesive films and incubated for 2 h at 37 °C. Regarding MST, a 110 µL-reaction mixture contained 0.5 mM or 1 mM of each compound, 30 µg/well MST in 50 mM Tris-HCl pH 8.0, and 10 mM dithiothreitol. After keeping the plate on ice for 30 min, the reaction was triggered by adding 1.5 mM sodium 3-mercaptopyruvate. The plate was sealed with vinyl adhesive films and incubated for 1 h at 37 °C. After the above mentioned incubation times, the CBS, CSE and MST reaction plates were kept on ice for 15 min. Then, 110 µl of 4% zinc acetate were dispensed to each well by punching a hole through the strip with a gas-tight Hamilton syringe. After 15-min incubation on ice, the films were removed and 15 µl of 60 mM NNDPD (3.6 mM final concentration) and 15 µl of 90 mM FeCl_3_ (5.4 mM final concentration) were dispensed to each well (final volume: 250 µl). The plate was incubated for 10 min at room temperature in the dark and the absorbance measured afterwards at 690 nm. Data were acquired using the plate reader Thermo Scientific Appliskan Multimode.

### Statistical Analysis

The Z’-factor was evaluated for each assay as described in^49^. Data were reported in the tables with their corresponding Z-score and coefficient of variation (CV). A |Z-score| threshold value of 3 was assumed to evaluate the effect of the screened compounds. All calculations were performed using Excel (Microsoft).

## Supplementary information

Supplementary information

## Data Availability

The datasets generated and/or analysed during the current study are available from the corresponding author on reasonable request.
