# Supplementary material for: Screening Pyridine Derivatives against Human Hydrogen Sulfide-synthesizing Enzymes by Orthogonal Methods
Source: Sci Rep. 2019 Jan 24;9:684. doi: 10.1038/s41598-018-36994-w (PMC6346012; doi:10.1038/s41598-018-36994-w)
Supplement: Supplementary file 1 — Supplementary information [file 41598_2018_36994_MOESM1_ESM.pdf]

Supplementary Information

Screening Pyridine Derivatives against Human Hydrogen Sulfide-synthesizing Enzymes by Orthogonal Methods

Karim Zuhra, Pedro M. F. Sousa, Giulia Paulini, Ana Rita Lemos, Zenta Kalme, Imants Bisenieks, Egils Bisenieks, Brigita Vigante, Gunars Duburs, Tiago M. Bandeiras, Luciano Saso, Alessandro Giuffrè, and João B. Vicente

. **Supplementary Figure S1.** Positive and negative controls for differential scanning fluorimetry thermal denaturation profiles of human H_2S-synthesizing enzymes
. **Supplementary Table S1.** Differential scanning fluorimetry analysis of the interaction between human tCBS and pyridine derivatives
. **Supplementary Figure S2.** Differential scanning fluorimetry thermal denaturation profiles of human tCBS incubated with pyridine derivatives
. **Supplementary Table S2.** Differential scanning fluorimetry analysis of the interaction between human CSE and pyridine derivatives
. **Supplementary Figure S3.** Differential scanning fluorimetry thermal denaturation profiles of human CSE incubated with pyridine derivatives
. **Supplementary Table S3.** Differential scanning fluorimetry analysis of the interaction between human MST and pyridine derivatives
. **Supplementary Figure S4.** Differential scanning fluorimetry thermal denaturation profiles of human MST incubated with pyridine derivatives
. **Supplementary Figure S5.** Surface plasmon resonance sensorgrams for human tCBS incubated with pyridine derivatives
. **Supplementary Figure S6.** Surface plasmon resonance sensorgrams for human CSE incubated with pyridine derivatives
. **Supplementary Figure S7.** Surface plasmon resonance sensorgrams for human MST incubated with pyridine derivatives
. **Supplementary Figure S8.** Surface plasmon resonance sensorgrams for human tCBS and CSE incubated with AOAA
. **Supplementary Table S4.** Effect of compounds on fluorimetric detection of H_2S produced by tCBS
. **Supplementary Table S5.** Effect of compounds on fluorimetric detection of H_2S released by GYY4137
. **Supplementary Figure S9.** Interaction between selected pyridine derivatives and the H_2S detecting fluorescent probe AzMc
. **Supplementary Table S6.** Effect of compounds on colorimetric detection of H_2S released by GYY4137

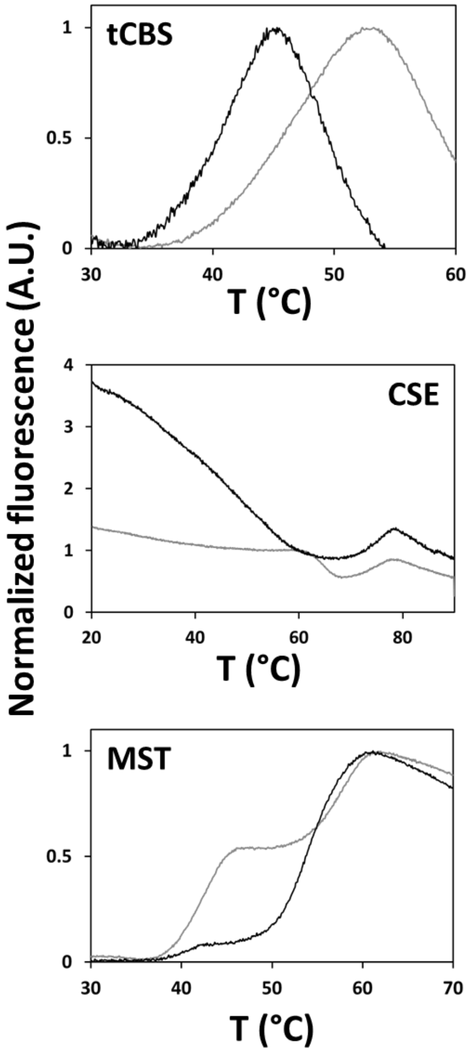

Supplementary Figure S1. Positive and negative controls for differential scanning fluorimetry thermal denaturation profiles of human H_2S-synthesizing enzymes. Thermal denaturation profiles of recombinant human cystathionine β-synthase (tCBS, *top panel*), cystathionine γ-lyase (CSE, *middle panel*) and 3-mercaptopyruvate sulfurtransferase (MST, *bottom panel*), obtained by differential scanning fluorimetry (DSF) in the absence (grey line; positive control) or presence (black line; negative control) of 200 μM aminooxyacetic acid (for tCBS and CSE) or 2 mM 3-mercaptopyruvate (for MST).

Supplementary Table S1 - Differential scanning fluorimetry analysis of the interaction between human tCBS and pyridine derivatives

	T_{m1} (*Z*’ factor: -1.58)				T_{m2} (*Z*’ factor: 0.08)				$T_{m'Ave'}$ (*Z*’ factor: -0.28)			
	**Mean (°C)**	**CV (%)**	**Z-score**	**ΔT_{m1} (°C)**	**Mean (°C)**	**CV (%)**	**Z-score**	**ΔT_{m2} (°C)**	**Mean (°C)**	**CV (%)**	**Z-score**	**$\Delta T_{m'Ave'}$ (°C)**
**Positive control (DMSO; *N* = 23)**	**42.3**	**4.0**	**---**	**---**	**48.4**	**1.5**	**---**	**---**	**45.6**	**1.8**	**---**	**---**
**Negative control (AOAA in DMSO; *N* = 25)**	**37.7**	**5.9**	**2.7**	**-4.6**	**42.2**	**2.8**	**8.5**	**-6.2**	**40.3**	**3.7**	**6.5**	**-5.3**
C1	42.6	4.7	-0.1	0.2	49.4	1.1	-1.3	1.0	46.5	1.8	-1.1	0.9
C2	39.5	3.9	1.6	-2.8	48.5	0.5	-0.2	0.1	44.6	2.6	1.2	-1.0
C3	40.7	3.4	0.9	-1.6	48.9	0.5	-0.7	0.5	45.5	0.4	0.2	-0.1
C4	40.0	4.5	1.4	-2.4	48.7	0.5	-0.5	0.4	45.3	1.5	0.4	-0.3
C5	41.8	1.8	0.3	-0.5	48.7	0.3	-0.4	0.3	45.9	0.0	-0.3	0.3
C6	40.8	4.7	0.9	-1.6	48.8	0.5	-0.6	0.4	45.7	0.6	-0.2	0.1
C7	41.6	0.8	0.5	-0.8	48.7	0.2	-0.4	0.3	45.6	1.1	0.0	0.0
C8	41.4	0.6	0.5	-0.9	48.8	0.6	-0.5	0.4	45.7	0.3	-0.2	0.1
C9	---	---	---	---	---	---	---	---	---	---	---	---
C10	---	---	---	---	---	---	---	---	---	---	---	---
C11	40.5	2.4	1.1	-1.8	48.7	0.5	-0.4	0.3	45.9	0.6	-0.3	0.3
C12	40.1	4.9	1.3	-2.2	48.8	0.5	-0.6	0.4	45.5	0.8	0.2	-0.1
C13	42.8	2.5	-0.3	0.5	49.2	0.5	-1.1	0.8	46.4	0.9	-1.0	0.8
C14	43.4	7.1	-0.6	1.0	49.4	1.5	-1.4	1.0	46.7	2.4	-1.3	1.1
C15	41.9	2.0	0.3	-0.5	48.7	0.5	-0.4	0.3	46.0	0.4	-0.5	0.4
C16	41.5	1.8	0.5	-0.9	48.7	0.4	-0.5	0.3	45.9	1.2	-0.3	0.3
C17	41.1	1.5	0.7	-1.2	48.6	0.2	-0.3	0.2	45.5	0.5	0.1	-0.1
C18	39.3	2.3	1.8	-3.1	48.4	1.6	-0.1	0.0	45.1	0.8	0.6	-0.5
C19	---	---	---	---	---	---	---	---	---	---	---	---
C20	39.7	4.0	1.6	-2.7	48.6	0.7	-0.3	0.3	45.5	1.3	0.1	-0.1
C21	40.9	1.9	0.8	-1.4	48.7	0.2	-0.5	0.4	46.0	0.2	-0.4	0.4
C22	39.8	3.7	1.5	-2.5	48.5	0.8	-0.1	0.1	45.2	0.8	0.5	-0.4
C23	---	---	---	---	---	---	---	---	---	---	---	---
C24	42.6	1.2	-0.2	0.3	48.9	0.4	-0.7	0.5	46.1	0.2	-0.6	0.5
C25	42.8	2.9	-0.3	0.4	49.2	0.7	-1.1	0.8	46.3	0.9	-0.8	0.7
C26	43.4	1.9	-0.6	1.0	49.5	0.5	-1.5	1.1	46.5	1.0	-1.1	0.9
C27	43.2	2.1	-0.5	0.8	48.9	0.3	-0.7	0.5	45.9	1.2	-0.4	0.3
C28	43.6	4.4	-0.8	1.3	49.6	1.2	-1.6	1.2	47.4	0.8	-2.1	1.8
C29	44.3	4.1	-1.2	2.0	49.8	1.2	-1.9	1.4	47.0	1.4	-1.7	1.4
C30	42.5	2.8	-0.1	0.1	48.8	1.1	-0.5	0.4	46.0	1.7	-0.5	0.4
C31	42.2	2.3	0.1	-0.2	48.4	0.4	0.0	0.0	45.9	1.4	-0.3	0.3

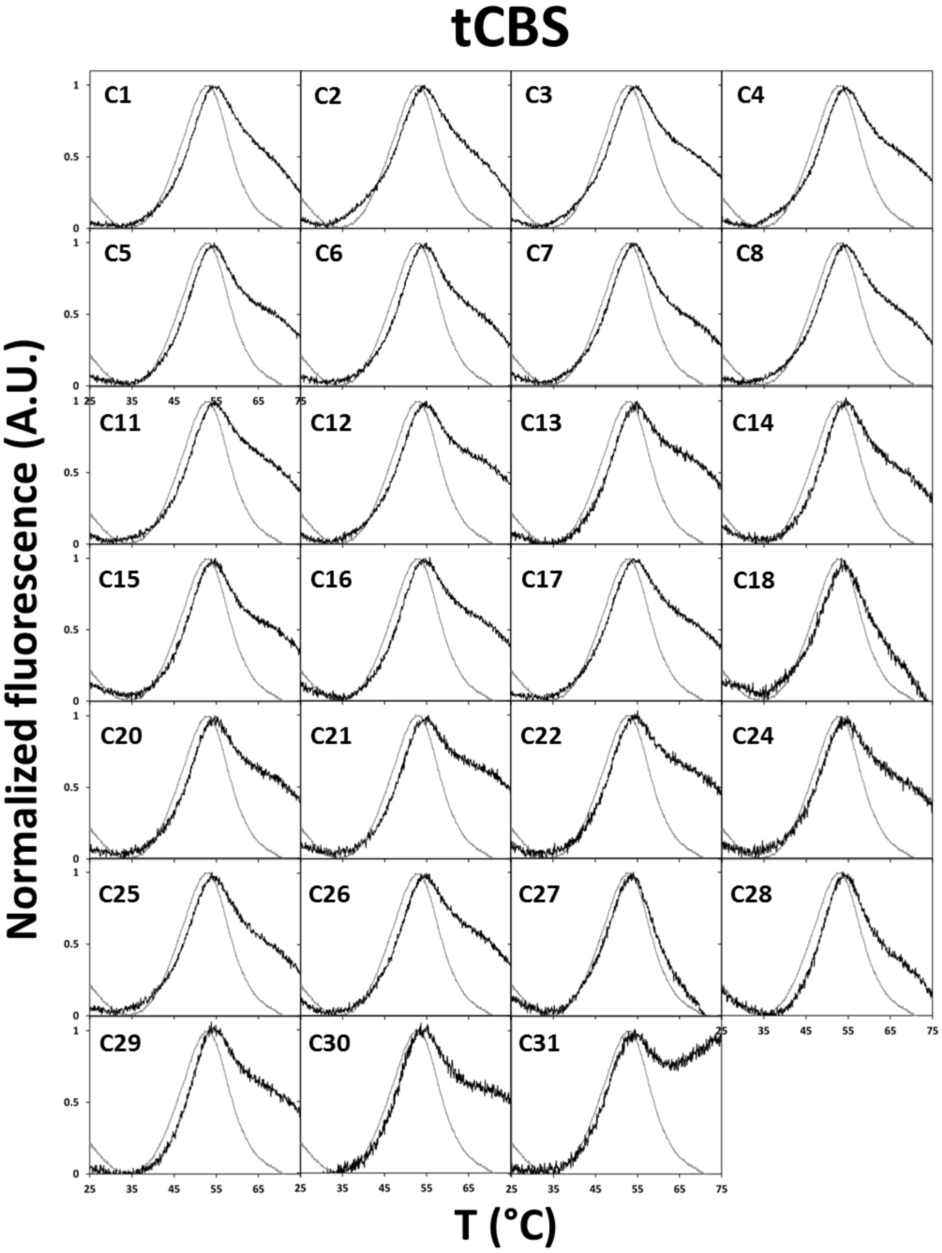

Supplementary Figure S2. Differential scanning fluorimetry thermal denaturation profiles of human tCBS incubated with pyridine derivatives. Experimental conditions as described in the Materials and Methods section.

Supplementary Table S2 - Differential scanning fluorimetry analysis of the interaction between human CSE and pyridine derivatives

	Ratio A (Z' factor: 0.53)			Ratio B (Z' factor: 0.31)		
	**Mean**	**CV (%)**	**Z-score**	**Mean**	**CV (%)**	**Z-score**
**Positive control (DMSO; *N* = 26)**	1.4	7.9	---	0.6	8.1	---
**Negative control (AOAA in DMSO; *N* = 26)**	3.8	7.1	**-22.1**	0.9	3.5	**-7.3**
C1	2.1	22.7	-6.2	0.4	2.9	3.7
C2	2.1	18.0	-6.3	0.4	7.1	3.9
C3	1.7	28.6	-2.4	0.4	5.1	4.0
C4	1.7	22.9	-2.6	0.4	1.2	2.9
C5	1.6	17.5	-2.1	0.4	2.9	3.7
C6	1.6	23.6	-2.1	0.4	5.5	3.4
C7	1.6	10.2	-2.0	0.4	8.0	4.2
C8	1.5	8.3	-1.5	0.4	8.2	3.6
C9	2.2	2.8	-7.2	0.4	6.8	3.8
C10	---	---	---	---	---	---
C11	1.4	3.7	0.2	0.4	1.7	2.9
C12	1.3	3.2	1.0	0.4	6.7	3.3
C13	1.2	6.4	1.8	0.4	5.7	2.8
C14	1.3	2.6	0.8	0.5	4.0	2.4
C15	1.4	10.8	-0.3	0.5	6.3	2.0
C16	1.4	4.4	-0.4	0.4	2.8	2.9
C17	1.7	21.0	-3.3	0.4	3.9	3.6
C18	---	---	---	---	---	---
C19	---	---	---	---	---	---
C20	1.3	3.5	1.0	0.5	2.0	1.6
C21	1.3	6.2	1.1	0.4	5.2	2.6
C22	1.3	10.3	0.4	0.5	1.2	1.6
C23	---	---	---	---	---	---
C24	1.4	7.1	-0.2	0.5	8.2	1.8
C25	1.3	2.4	0.5	0.4	5.9	2.8
C26	1.4	4.1	-0.1	0.4	4.4	3.2
C27	1.3	9.7	0.5	0.6	1.0	-1.4
C28	1.3	13.1	0.5	0.5	4.9	2.5
C29	1.2	6.7	2.1	0.6	1.8	-1.5
C30	1.0	2.0	3.2	0.7	1.4	-3.8
C31	1.0	4.6	3.6	0.8	1.2	-4.0

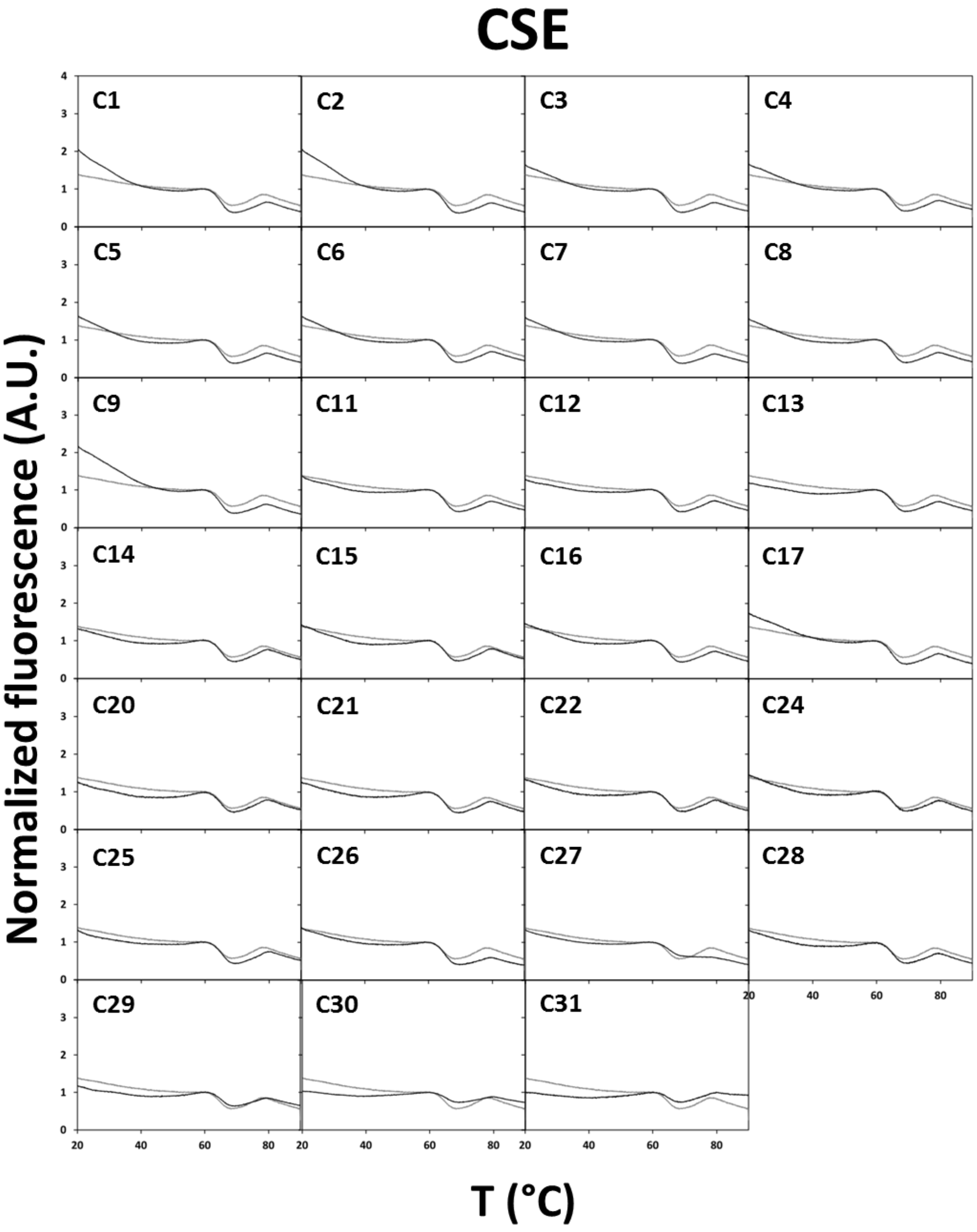

Supplementary Figure S3. Differential scanning fluorimetry thermal denaturation profiles of human CSE incubated with pyridine derivatives. Experimental conditions as described in the Materials and Methods section.

Supplementary Table S3 - Differential scanning fluorimetry analysis of the interaction between human MST and pyridine derivatives

	**Frac (Z' factor: 0.50)**			**T_{m1} (Z' factor: -0.32)**				**T_{m2} (Z' factor: 0.68)**				**$T_{m'Ave'}$ (Z' factor: -0.06)**			
	**Mean**	**CV (%)**	**Z-score**	**Mean (°C)**	**CV (%)**	**Z- score**	**ΔT_{m1} (°C)**	**Mean (°C)**	**CV (%)**	**Z-score**	**ΔT_{m2} (°C)**	**Mean (°C)**	**CV (%)**	**Z-score**	**$\Delta T_{m'Ave'}$ (°C)**
**Positive control (*N* = 31)**	**0.54**	**10.9**	**---**	**41.9**	**0.6**	**---**	**0**	**57.2**	**0.3**	**---**	**0**	**49.2**	**2.4**	**---**	**0**
**Negative control (*N* = 28)**	**0.05**	**46.0**	**8.3**	**40.4**	**1.0**	**6.0**	**-1.5**	**54.1**	**0.3**	**19.5**	**-3.1**	**53.5**	**0.6**	**-3.6**	**4.3**
C1	0.51	9.6	0.5	41.3	0.3	2.4	-0.6	56.6	0.2	4.0	-0.6	48.7	1.6	0.4	-0.5
C2	0.48	7.6	1.0	40.6	0.4	5.2	-1.3	56.5	0.3	4.2	-0.7	48.8	1.2	0.3	-0.4
C3	0.53	5.6	0.2	42.1	0.2	-0.6	0.2	56.7	0.2	3.5	-0.5	48.9	0.9	0.2	-0.3
C4	0.52	6.7	0.3	41.8	0.2	0.6	-0.2	56.8	0.2	2.7	-0.4	48.9	0.9	0.2	-0.3
C5	0.59	3.8	-0.7	42.2	0.5	-1.3	0.3	56.6	0.3	4.0	-0.6	48.2	0.4	0.8	-1.0
C6	0.55	3.1	-0.1	42.3	0.2	-1.5	0.4	56.8	0.2	2.4	-0.4	48.8	0.5	0.3	-0.3
C7	0.57	3.5	-0.5	41.9	0.5	-0.1	0.0	56.7	0.2	3.4	-0.5	48.3	0.6	0.8	-0.9
C8	0.58	6.0	-0.7	42.2	0.3	-1.2	0.3	56.8	0.1	2.9	-0.4	48.3	1.0	0.7	-0.9
C9	0.53	2.3	0.2	42.1	0.5	-0.6	0.2	56.4	0.2	5.3	-0.8	48.8	0.3	0.3	-0.4
C10	0.49	14.1	0.9	41.9	0.2	-0.1	0.0	56.4	0.0	5.4	-0.8	49.3	2.1	-0.1	0.1
C11	0.51	6.2	0.5	42.1	0.1	-0.9	0.2	56.6	0.1	3.7	-0.6	49.2	0.9	0.0	0.0
C12	0.54	3.9	0.0	42.4	0.1	-1.9	0.5	56.7	0.1	3.3	-0.5	48.9	0.7	0.2	-0.3
C13	0.59	4.2	-0.8	42.4	0.4	-1.9	0.5	56.6	0.1	3.6	-0.6	48.2	0.8	0.8	-1.0
C14	0.54	5.2	0.0	42.2	0.4	-1.0	0.3	56.7	0.2	3.0	-0.5	48.8	0.7	0.3	-0.4
C15	0.56	3.8	-0.2	42.1	0.3	-0.6	0.2	56.8	0.1	2.7	-0.4	48.6	0.8	0.5	-0.6
C16	0.56	9.4	-0.3	42.3	0.1	-1.5	0.4	56.7	0.1	3.3	-0.5	48.6	1.5	0.5	-0.6
C17	0.54	2.2	0.1	42.3	0.1	-1.4	0.4	56.6	0.1	4.0	-0.6	48.9	0.3	0.2	-0.3
C18	0.57	5.6	-0.5	42.3	0.1	-1.5	0.4	56.6	0.0	3.6	-0.6	48.4	0.9	0.6	-0.8
C19	---	---	---	---	---	---	---	---	---	---	---	---	---	---	---
C20	0.56	7.0	-0.2	42.1	0.1	-0.7	0.2	56.7	0.3	3.5	-0.5	48.6	1.3	0.5	-0.6
C21	0.57	6.7	-0.4	42.2	0.7	-1.2	0.3	56.5	0.1	4.6	-0.7	48.4	1.4	0.6	-0.8
C22	0.53	4.5	0.2	42.3	0.4	-1.7	0.4	56.7	0.4	3.2	-0.5	49.1	0.9	0.1	-0.1
C23	---	---	---	---	---	---	---	---	---	---	---	---	---	---	---
C24	0.50	5.8	0.6	42.4	1.6	-1.9	0.5	56.5	0.1	4.2	-0.7	49.4	0.7	-0.2	0.2
C25	0.57	4.2	-0.5	42.0	0.2	-0.4	0.1	56.8	0.1	2.3	-0.4	48.3	0.7	0.7	-0.8
C26	0.60	6.1	-1.0	42.4	0.1	-1.8	0.5	56.7	0.2	3.4	-0.5	48.1	1.1	0.9	-1.1
C27	0.61	7.8	-1.1	42.5	0.4	-2.1	0.5	56.7	0.2	3.0	-0.5	48.1	1.5	0.9	-1.1
C28	0.56	5.2	-0.3	42.3	0.5	-1.6	0.4	56.7	0.1	3.2	-0.5	48.6	0.8	0.5	-0.5
C29	0.53	11.0	0.3	42.3	0.5	-1.7	0.4	56.3	0.5	5.9	-0.9	48.9	1.3	0.2	-0.2
C30	0.55	0.4	-0.2	40.8	0.2	4.4	-1.1	56.4	0.4	4.8	-0.8	47.8	0.3	1.1	-1.4
C31	0.43	15.5	1.9	40.2	0.1	6.7	-1.7	57.0	0.3	1.2	-0.2	49.8	2.5	-0.5	0.6

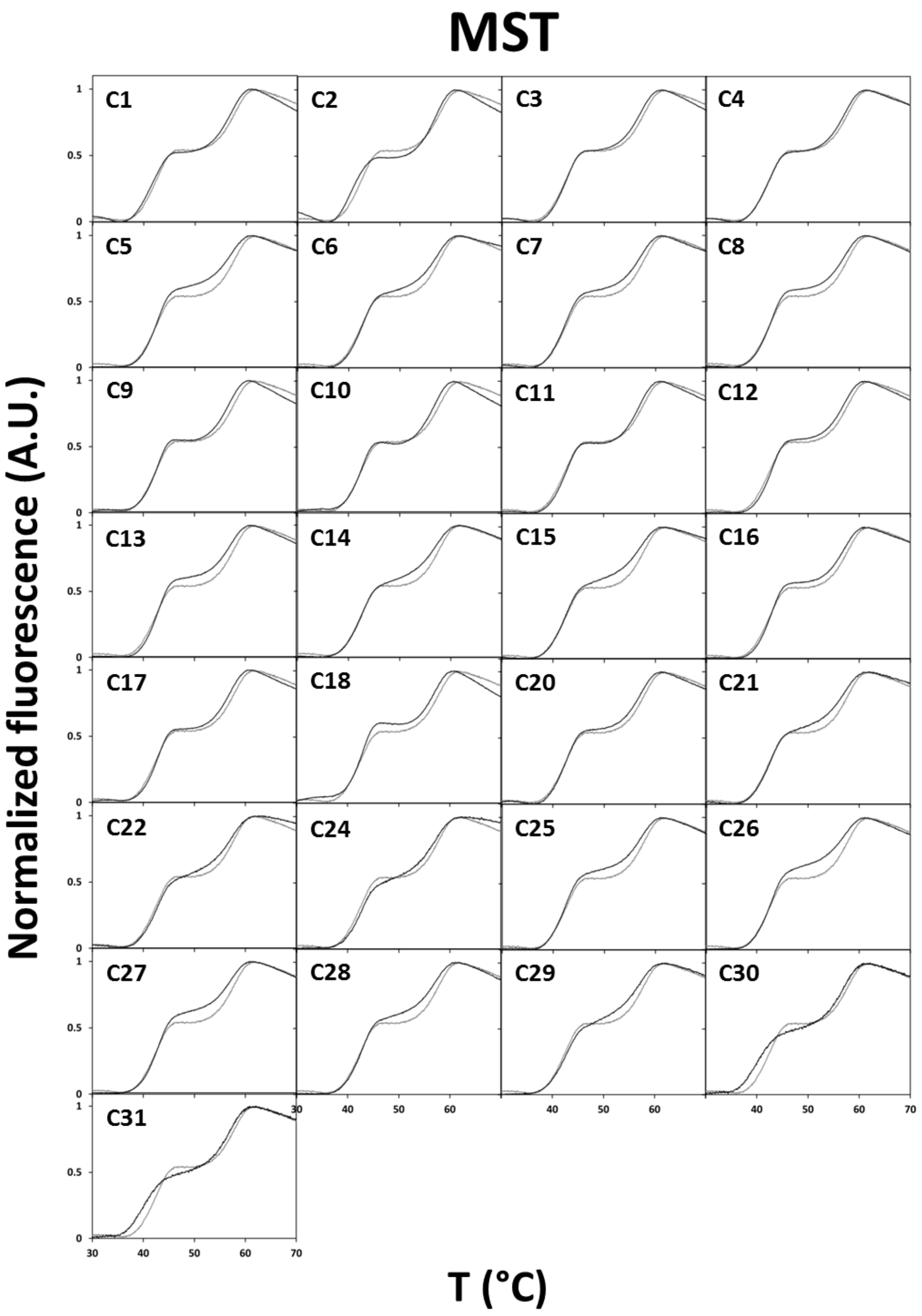

Supplementary Figure S4. Differential scanning fluorimetry thermal denaturation profiles of human MST incubated with pyridine derivatives. Experimental conditions as described in the Materials and Methods section.

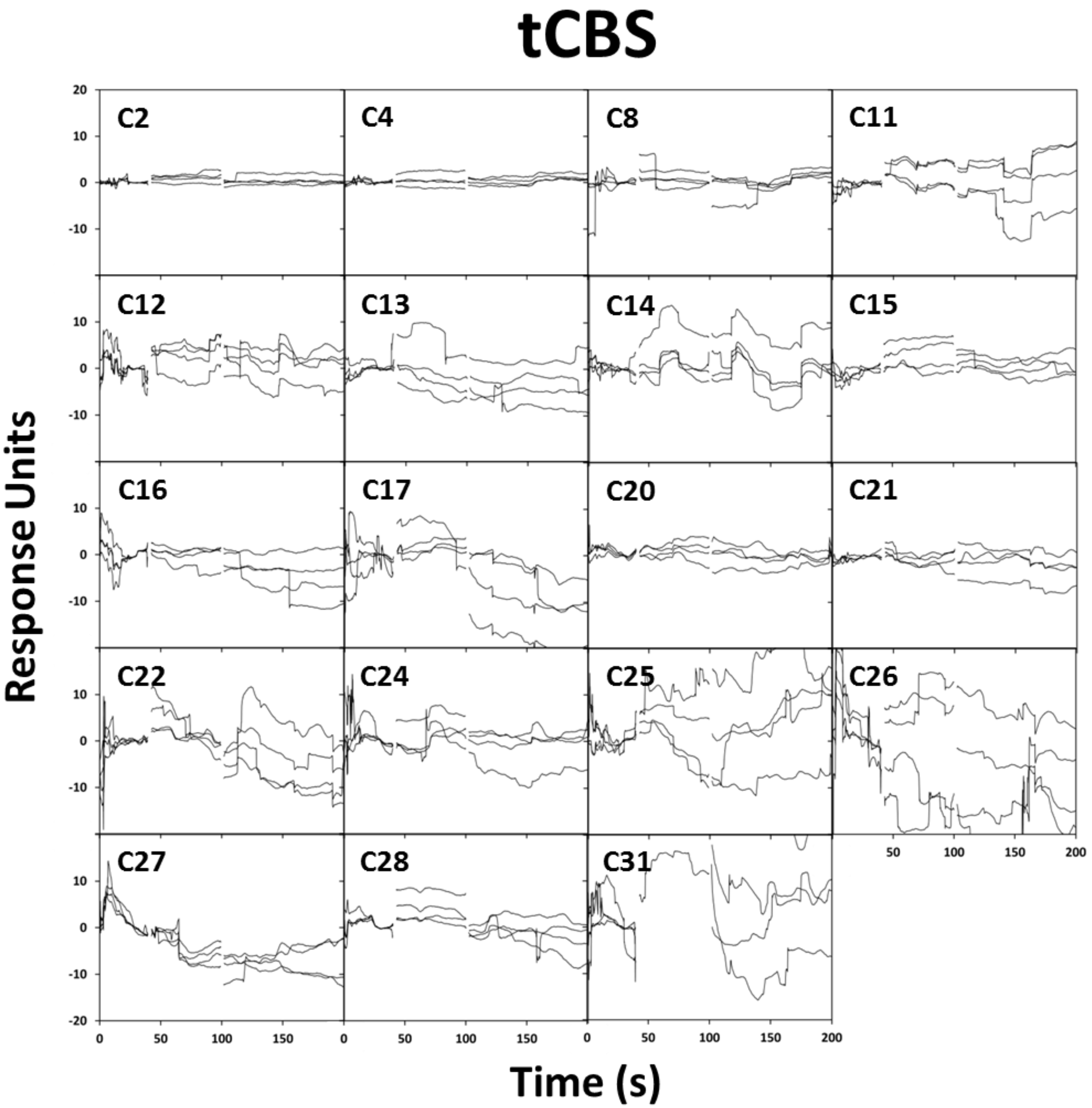

Supplementary Figure S5. Surface plasmon resonance sensorgrams for human tCBS incubated with pyridine derivatives. Experimental conditions as described in the Materials and Methods section.

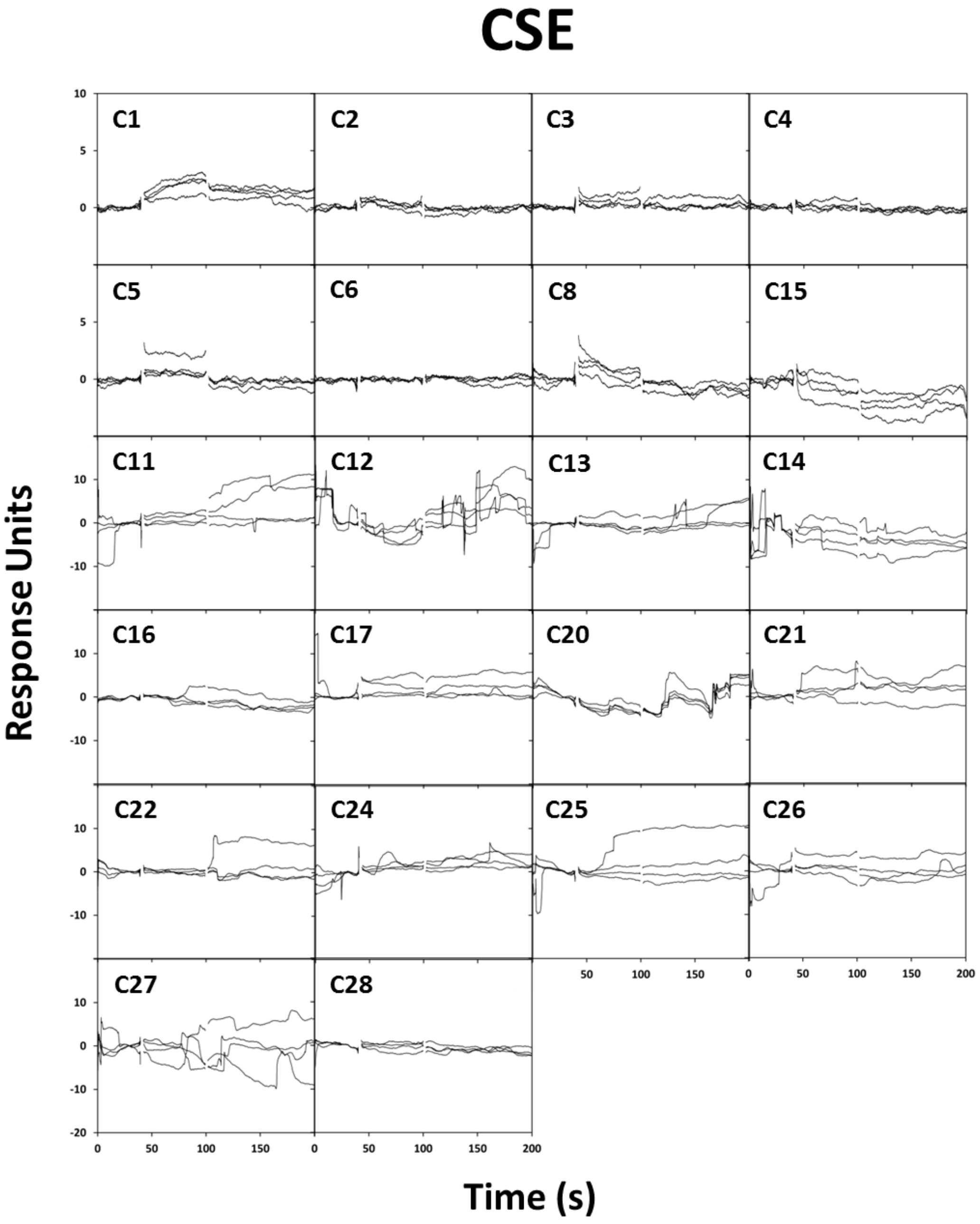

Supplementary Figure S6. Surface plasmon resonance sensorgrams for human CSE incubated with pyridine derivatives. Experimental conditions as described in the Materials and Methods section.

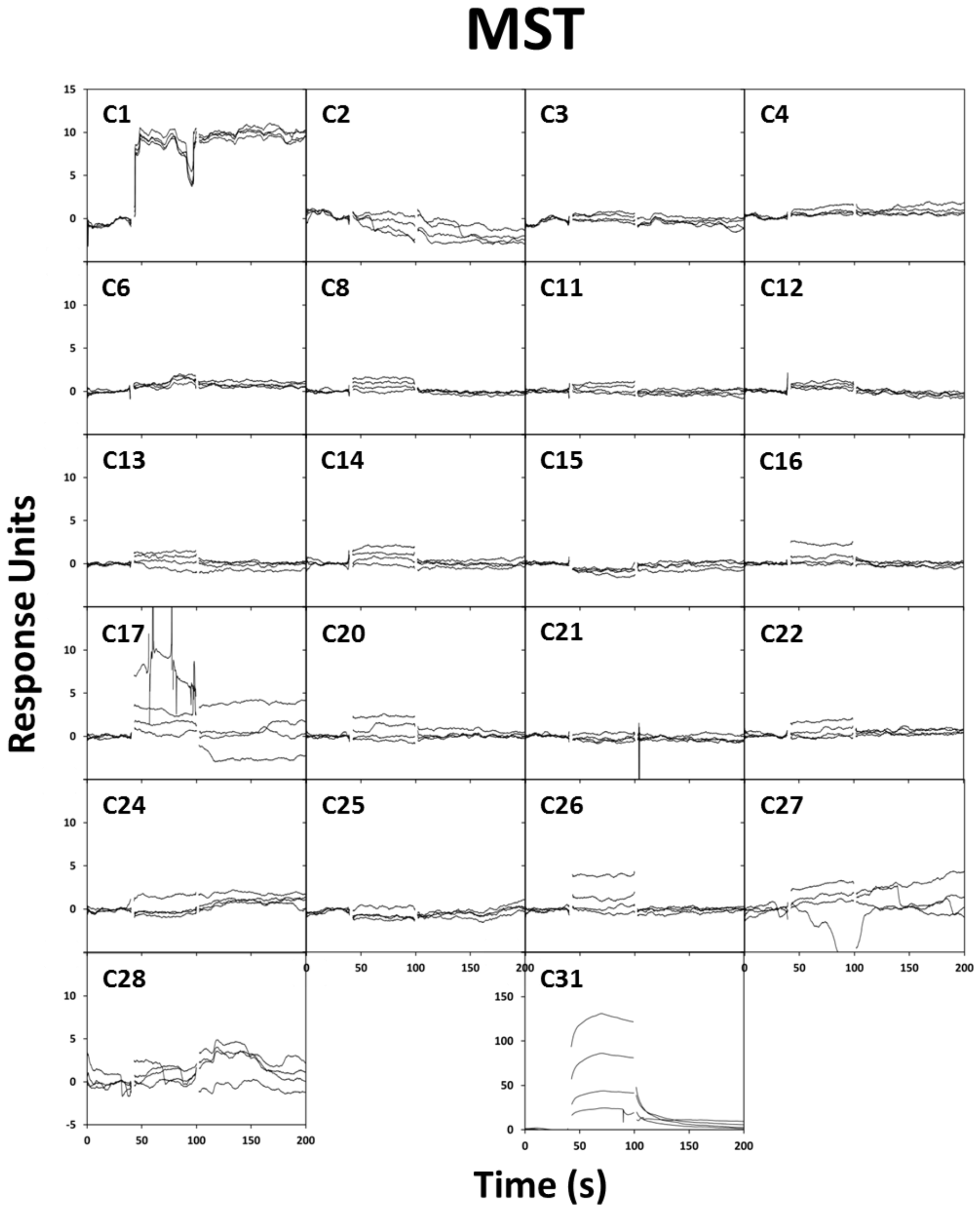

Supplementary Figure S7. Surface plasmon resonance sensorgrams for human MST incubated with pyridine derivatives. Experimental conditions as described in the Materials and Methods section.

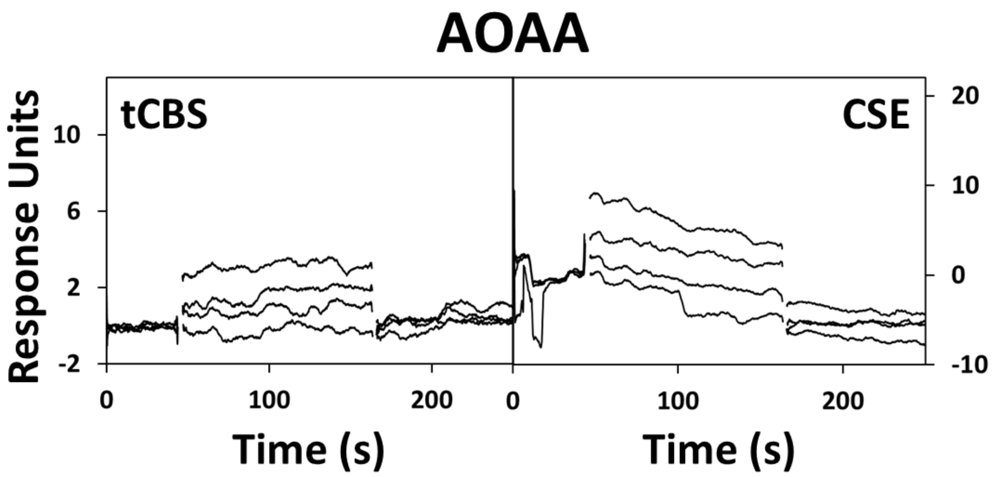

Supplementary Figure S8. Surface plasmon resonance sensorgrams for human tCBS and CSE incubated with AOAA. Experimental conditions as described in the Materials and Methods section.

Supplementary Table S4 - Effect of compounds on fluorimetric detection of H_2S produced by tCBS. Experiments aimed at evaluating the possible effect of pyridine derivatives on the H_2S-synthesizing activity of tCBS, making use of the H_2S-selective fluorescent probe AzMC. Experimental conditions as described in the Materials and Methods section. Unless otherwise indicated, data represent mean values of triplicate determinations. Compounds were tested at a final concentration of 200 µM.

	Frac (Z' factor: 0.78)		
	**Fluorescence (%)**	**CV (%)**	**Z-score**
**Positive control (*N* = 16)**	**100.0**	**6.6**	**---**
**Negative control (*N* = 16)**	**2.8**	**14.2**	**14.5**
**C2**	52.8	4.9	7.0
**C3**	49.4	6.9	7.6
**C5**	46.6	5.6	8.0
**C8**	31.2	4.9	10.3
**C11**	50.2	78.1	7.4
**C14**	52.6	20.3	7.1
**C15**	43.0	14.6	8.5
**C16**	28.2	6.4	10.7
**C17**	40.9	4.4	8.8
**C18**	46.7	6.8	8.0
**C21**	37.6	13.1	9.3
**C23**	34.9	4.2	9.7
**C26**	41.9	7.2	8.7
**C28**	51.3	7.4	7.3
**C30**	46.5	7.4	8.0
**C31**	49.9	5.3	7.5

Supplementary Table S5 - Effect of compounds on fluorimetric detection of H_2S released by GYY4137. Experiments showing interference of pyridine derivatives with detection of GYY4137-released H_2S by the selective fluorescent probe AzMC. Experimental conditions as described in the Materials and Methods section. Unless otherwise indicated, data represent mean values of triplicate determinations. Compounds were tested at a final concentration of 500 µM.

	Frac (Z' factor: 0.85)		
	**Fluorescence (%)**	**CV (%)**	**Z-score**
**Positive control (N = 16)**	100.00	7.9	---
**Negative control (N = 16)**	12.3	6.5	11.9
**C2**	30.9	8.1	8.7
**C3**	38.9	5.0	7.7
**C5**	27.7	6.8	9.1
**C8**	16.6	6.2	10.5
**C11**	65.7	9.7	4.3
**C14**	20.5	13.9	10.1
**C15**	27.3	11.1	9.2
**C16**	24.0	5.4	9.6
**C17**	22.6	23.1	9.8
**C18**	31.1	9.6	8.7
**C21**	20.8	9.1	10.0
**C23**	15.9	23.0	10.6
**C26**	11.7	13.2	11.2
**C28**	22.6	17.3	9.8
**C30**	32.3	6.7	8.6
**C31**	31.7	7.0	8.6

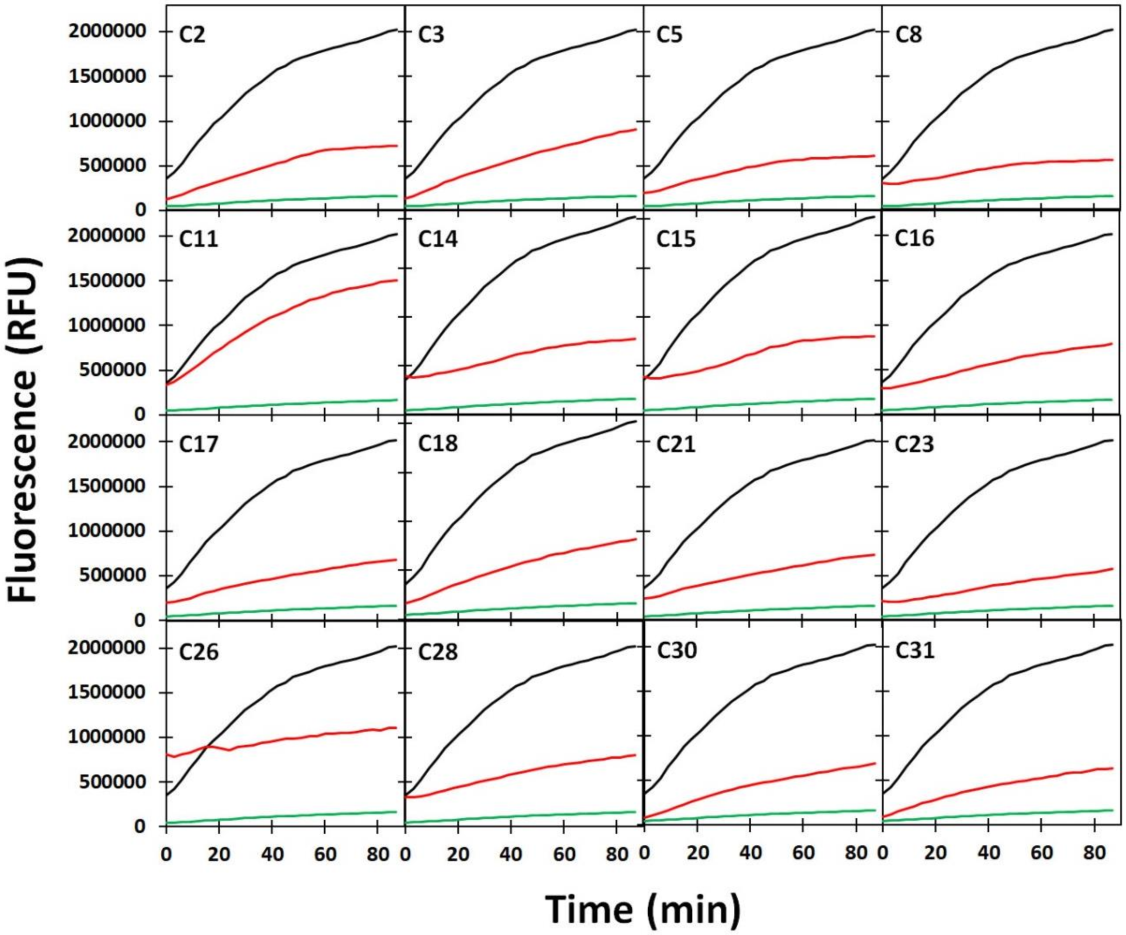

Supplementary Figure S9. Interaction between selected pyridine derivatives and the H_2S detecting fluorescent probe AzMc. H_2S release by GYY 4137 detected by the fluorescent probe AzMc in the absence (black lines) and presence (red lines) of pyridine derivatives. Green line, blank control lacking GYY 4137. Experimental conditions as described in the Materials and Methods section.

Supplementary Table S6 - Effect of compounds on colorimetric detection of H_2S released by GYY4137. Experiments showing no interference of pyridine derivatives with detection of GYY4137-released H_2S by the methylene blue method. Experimental conditions as described in the Materials and Methods section. Unless otherwise indicated, data represent mean values of triplicate determinations. The asterisk denotes the compounds tested at 500 µM instead of 1 mM final concentration.

	**Frac (Z' factor: 0.84)**		
	**Absorbance (%)**	**CV (%)**	**Z-score**
**Positive control (*N* = 9)**	100,0	7,5	**---**
**Negative control (*N* = 9)**	4,3	2,9	12.8
C1*	88.7	8.3	1.5
C2	91.7	3.6	1.1
C3	91.7	1.4	1.1
C4	92.8	2.8	1.0
C5	92.9	5.1	0.9
C6*	103.1	3.0	-0.4
C7	100.9	4.7	-0.1
C8	101.7	1.3	-0.2
C9	96.9	5.3	0.4
C10	100.8	2.6	-0.1
C11	82.7	6.0	2.3
C12*	100.2	16.5	0.0
C13	102.7	12.5	-0.4
C14	93.6	9.6	0.9
C15	95.0	9.6	0.7
C16	94.4	13.9	0.8
C17	101.4	0.5	-0.2
C18	103.6	0.8	-0.5
C19	104.0	2.5	-0.5
C20*	91.7	2.2	1.1
C21*	93.3	5.7	0.9
C22	98.6	5.8	0.2
C23	99.0	4.9	0.1
C24*	94.1	2.9	0.8
C25	97.1	7.1	0.4
C26	96.2	3.5	0.5
C27	96.1	2.0	0.5
C28	95.6	1.4	0.6
C29	94.9	3.0	0.7
C30	99.1	7.1	0.1
C31*	96.7	2.8	0.4
